# Curcumin induces multiple signaling pathways leading to vascular smooth muscle cell senescence

**DOI:** 10.1007/s10522-019-09825-2

**Published:** 2019-08-01

**Authors:** Wioleta Grabowska, Grażyna Mosieniak, Natalia Achtabowska, Robert Czochara, Grzegorz Litwinienko, Agnieszka Bojko, Ewa Sikora, Anna Bielak-Zmijewska

**Affiliations:** 1 Nencki Institute of Experimental Biology, Polish Academy of Sciences, 3 Pasteur St, 02-093 Warsaw, Poland; 2 Faculty of Chemistry, Warsaw University of Technology, 3 Noakowskiego St, 00-664 Warsaw, Poland; 3 Faculty of Chemistry, University of Warsaw, 1 Pasteur St, 02-093 Warsaw, Poland

**Keywords:** Curcumin, VSMCs, Senescence, ATM, AMPK, β-glucuronidase

## Abstract

Curcumin, a phytochemical present in the spice named turmeric, and one of the promising anti-aging factors, is itself able to induce cellular senescence. We have recently shown that cells building the vasculature senesced as a result of curcumin treatment. Curcumin-induced senescence was DNA damage-independent; however, activation of ATM was observed. Moreover, neither increased ROS production, nor even ATM were indispensable for senescence progression. In this paper we tried to elucidate the mechanism of curcumin-induced senescence. We analyzed the time-dependence of the level and activity of numerous proteins involved in senescence progression in vascular smooth muscle cells and how inhibition p38 or p38 together with ATM, two proteins involved in canonical signaling pathways, influenced cell senescence. We showed that curcumin was able to influence many signaling pathways of which probably none was dominant and sufficient to induce senescence by itself. However, we cannot exclude that the switch between initiation and progression of senescence is the result of the impact of curcumin on signaling pathways engaging AMPK, ATM, sirtuin 1 and p300 and on their reciprocal interplay. Cytostatic concentration of curcumin induced cellular stress, which exceeded the adaptive response and, in consequence, led to cellular senescence, which is triggered by time dependent activation of several signaling pathways playing diverse roles in different phases of senescence progression. We also showed that activity of β-glucuronidase, the enzyme involved in deconjugation of the main metabolites of curcumin, glucuronides, increased in senescent cells. It suggests a possible local elevation of curcumin concentration in the organism.

**Electronic supplementary material:**

The online version of this article (10.1007/s10522-019-09825-2) contains supplementary material, which is available to authorized users.

## Introduction

Curcumin is a natural compound, a supplement of the diet and a promising anti-aging factor with documented activity in elongation of lifespan of animal models (Liao et al. 2011; Lee et al. 2010; Soh et al. 2013; Shen et al. 2013). However, curcumin itself can induce cellular senescence, as we have documented for cancer and normal cells (Mosieniak et al. 2012; Grabowska et al. 2015). It is believed that cellular senescence is involved in aging and age-related diseases. Senescent cells have a negative impact on tissue functions and regenerative potential. Furthermore, through increased production of mediators of inflammation, they influence the whole organism. Removal of senescent cells visibly improved the physical functioning of mice (Baker et al. 2011; Baker et al. 2016). On the other hand, senescence is essential for proper regeneration and protection from some diseases (tumor, atherosclerosis) (Muñoz-Espín and Serrano 2014). Thus, it cannot be excluded that cell senescence induced by curcumin can play a beneficial role. Indeed, it has been shown that curcumin-induced senescence of hepatic stellate cells (HSC) protects from liver fibrosis (Jin et al. 2017) and, in the case of cancer-associated fibroblasts (CAF), defends against cancer invasion (Hendrayani et al. 2013). Our recent study revealed that, in vitro, curcumin is able to induce senescence in cells building the vasculature (Grabowska et al. 2015), however, the impact of this process on aging of the organism is an open question.

In this study we intended to elucidate the mechanism(s) of senescence induced by curcumin. So far we have shown that curcumin-induced senescence of vascular smooth muscle cells (VSMCs) is DNA damage-independent and that neither ROS production nor ATM activation (the key component of DNA damage response pathway) are essential for senescence progression (Grabowska et al. 2015). We observed increased level and activity of p38, which suggested that senescence could be triggered by upregulation of this protein. However, the exact mechanism of curcumin-induced senescence still remains unresolved. In this study we tried to identify the cause and consequences of curcumin-induced senescence. To this aim, we analyzed changes in the activity of selected signaling pathways within a short time (up to 24 h) and between 1 and 7 days of curcumin treatment. Our analysis included: AMPK signaling pathway (protein responsible for energy homeostasis), ATR (protein activated in response to DNA, single strand breaks) and p300 (acetyltransferase responsible for acetylation of histones and regulation of gene expression, the level/activity of which shows age-dependent changes). Our results suggest that more than one signaling pathway is activated by curcumin and that the cell fate, i.e. senescence, depends on the interplay between many signaling pathways triggered by cell response to stress conditions. We have also shown that the level of β-glucuronidase, the enzyme involved in the reduction of curcumin glucuronides, the main metabolite of curcumin detected in the serum, increased in senescent cells. This can suggest elevated local concentration of free curcumin. Such increased concentration could have some adverse effect on the neighboring, non-senescent cells.

## Materials and methods

### Reagents

Curcumin (C1386) was from Cayman (Ann Arbor, USA); dimethyl sulfoxide (DMSO) (D4540), doxorubicin and DAPI were purchased from Sigma-Aldrich (St. Louis, USA); BSA was from BioShop (Burlington, Canada).

### Culture of vascular smooth muscle cells (VMSCs)

Human VMSCs were purchased from ATCC or from Lonza and cultured as described before (Bielak-Zmijewska et al. 2014). Cells used in experiments were treated with curcumin or doxorubicin at passages between 7 and 9 (young cells). Depending on the donor, cells underwent 18 to 30 passages. Cells were considered as replicatively senescent when the percentage of senescent cells in the whole cell population exceeded 80%. The efficacy of doxorubicin- or curcumin-dependent induction of senescence was almost 100%. Curcumin was dissolved in DMSO and the concentration of DMSO in cell culture did not exceed 0.1%. The HCT116 human colon cancer cell line was kindly provided by Dr. Bert Vogelstein (Johns Hopkins University, Baltimore, MD). Cells were grown in McCoy’s medium supplemented with 10% fetal bovine serum.

### Estimation of senescence associated-β-galactosidase activity

Detection of senescence associated-β-galactosidase (SA-β-gal) activity was performed according to Dimri et al. (1995). Cells were analyzed in a light microscope and counted (100 or more cells). The % of SA-β-gal-positive cells is shown.

### Cell cycle analysis

DNA content analysis was performed as described in Korwek et al. (2012). Briefly, DNA was stained with PI solution and 10,000 cells were analyzed using the FACSCalibur Becton–Dickinson flow cytometer and the CellQuestPro software.

### Western blotting analysis

Whole cell protein extracts were prepared according to Laemmli (1970). The primary antibodies used were: anti-ATM (1:500), anti-phospho-ATM Ser1981 (1:500), anti-p300 (1:500) (Abcam, Cambridge, UK), anti-GAPDH (1:50000) (Millipore, Darmstadt, Germany); anti-p21WAF1/Cip1 (1:500) (Sigma-Aldrich, St. Louis, USA); anti-p53 (1:500), (Santa Cruz Biotechnology, Santa Cruz, USA); anti-ATR (1:500), anti-phospho-ATR Ser428 (1:500), anti-phospho-p53 Ser15 (1:250), anti-acetyl-p53 Lys382 (1:200), anti-SIRT1 (1:250), anti-phospho-SIRT1 Ser47 (1:250), anti-p38 MAPK (1:500), anti-phospho-p38 MAPK Thr180/Tyr182 (1:500), anti-phospho-MAPKAPK-2 Thr334 (1:500), anti-AMPKα (1:500), anti-phospho-AMPKα Thr172 (1:1000), anti-ACC (1:500), anti-phospho-ACC Ser79 (1:1000), anti-mTOR (1:500), anti-phospho-mTOR Ser2448 (1:500), anti-phospho-S6 Ser235/236 (1:1000) (Cell Signaling Technology, Denvers, USA), anti-Rb (1:250) (NeoMarkers, Fremont, USA). The respective proteins were detected after incubation with one of the horseradish peroxidase-conjugated secondary antibodies (1:2000) (Dako, Glostrup, Denmark), using an ECL system (Thermo Scientific, Rockford, USA), according to the manufacturer’s instructions.

### Silencing of ATM

To downregulate *ATM* expression, cells were transfected with 30 nM siRNA against *ATM* (siATM) or negative siRNA (Thermo Fisher Scientific, Waltham, USA) using Lipofectamine2000 (Thermo Fisher Scientific, Waltham, USA). Transfection was performed according to the manufacturer’s protocol. About 24 h after transfection medium was replaced with fresh one. Cells with silenced *ATM* were treated with p38 inhibitor and/or curcumin as described below.

### Inhibition of p38 kinase

To inhibit p38 kinase cells were treated with p38 MAPK inhibitor SB203580 (Calbiochem, Merk Millipore, Darmstadt, Germany) about 24 h after seeding or 48 h after transfection with siATM or negative siRNA. The inhibitor was added 20 min prior to curcumin treatment. Cells were cultured up to 7 days and medium was replaced with fresh one 3 days after treatment and supplemented with SB203580 again.

### Measurement of β-glucuronidase activity

The activity of β-glucuronidase was measured using BioVision Assay Kit (BioVision Incorporated, Milpitas, CA, USA). The assay was performed following manufacturer’s protocol. Briefly, cells were trypsinized, centrifuged and lyzed in Assay Buffer. Lysates were centrifuged (10,000×*g*, 5 min, 4 °C) and supernatants were collected. 5 µl of supernatants, along with the positive and negative control, as well as standards, were transferred into 96-well plates. The Assay Buffer and the Substrate were added and fluorescence was measured immediately (Ex/Em = 330/450 nm) and then every 5 min for 1 h at 37 °C using an Infinite M1000 PRO microplate reader (Tecan). Activity of β-glucuronidase was calculated according to equation:

β-glucuronidase activity [µU/ml] = B/(Δt × V)

where, B—amount of the reaction product obtained in Δt, Δt—reaction time (when the linear increase of the product was observed), V—sample volume added to the reaction.

The results were normalized according to the number of harvested cells.

### Synthesis of curcumin glucuronides

Mono-(β-d-glucopyranosiduronic acid)-curcumin (curcumin monoglucuronide) was prepared by the 5-step method described by Choudhury et al. (2015) with modification of the last step, when mono-[methyl 2,3,4-tri-O-acetyl-β-d-glucopyranosiduronate]-curcumin was converted into mono-(β-d-glucopyranosiduronic acid)-curcumin as proposed by Pal et al. (2014). The final product was purified by column chromatography on silica gel eluted with chloroform–methanol. The intermediate compounds and the final product were characterized by 1H and 13C NMR (final product was additionally analyzed by LRMS). The detailed description is in the Online Resource.

### Statistical analysis

Analysis of normality of samples distribution was performed using Shapiro–Wilk test. Statistical analysis for SA-β-gal meets the conditions of normality and was performed using the 2-tailed Student/t/test. The distribution of densitometry data was not normal and therefore statistical analysis was performed by ANOVA test. The results for β-glucuronidase were analyzed in GraphPad Prism8 using one-way ANOVA with post hoc testing using a Dunnett’s multiple comparison test and results for curcumin glucuronide impact using ANOVA, followed by Tukey’s honestly significant difference (HSD) test. Data are presented as a mean ± SD. A value of p < 0.05 was considered statistically significant (*p < 0.05, **p < 0.01, ***p < 0.001). All graphs show the mean results from at least three independent experiments.

## Results

### Involvement of canonical p53/p21 and Rb –dependent signaling pathways in curcumin-treated cells

**Fig. 1:**
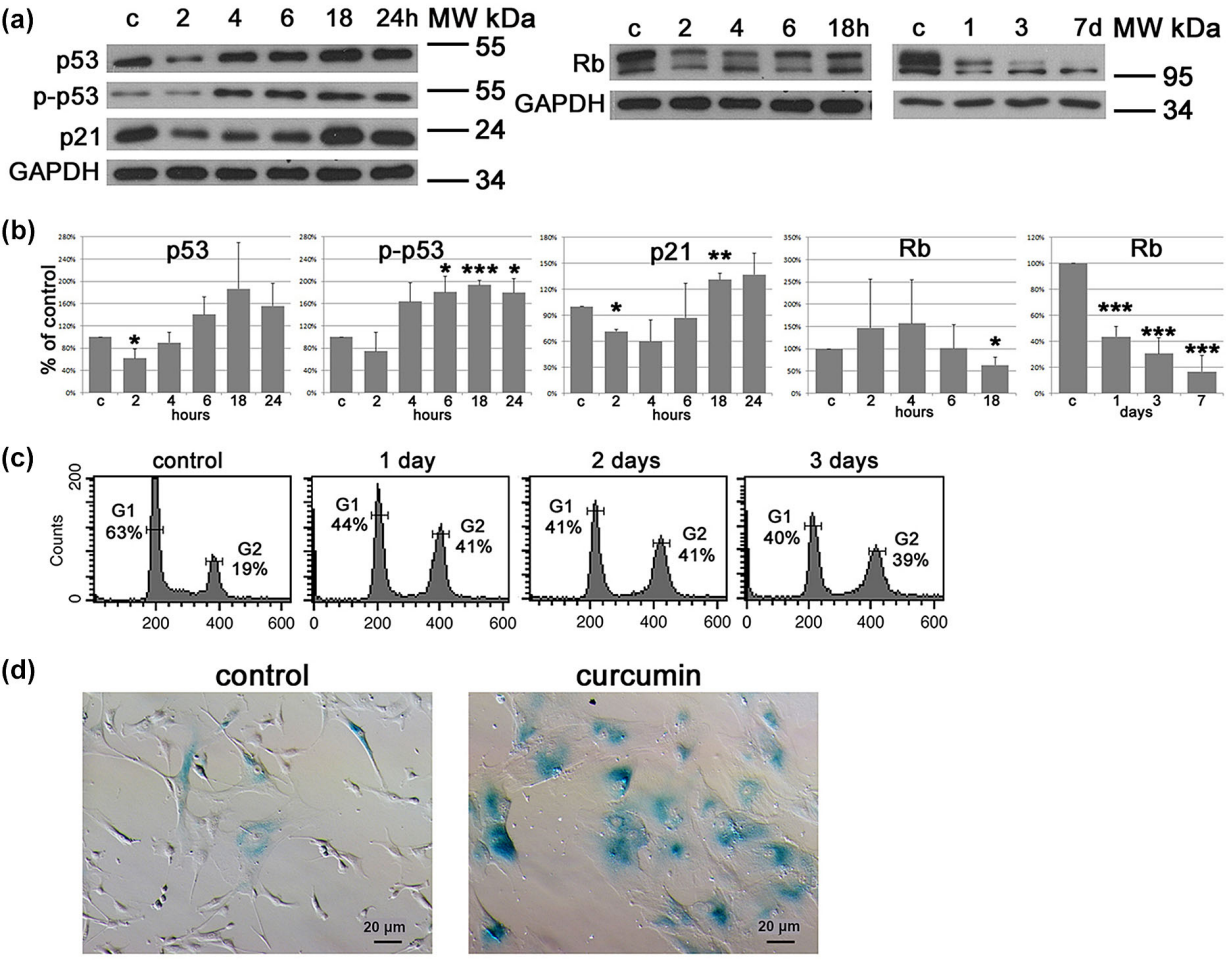
Analysis of proteins involved in the cell cycle arrest and of selected markers associated with cellular senescence. **a** Western blot analysis of Rb, p53, p-p53 and p21. GAPDH served as a loading control. Western blots representative for at least three independent experiments are shown. c—control, untreated cells. **b** Densitometric analysis of proteins analyzed by WB, mean ± SD from at least three independent experiments, *p < 0.05, **p < 0.01, ***p < 0.001. **c** Analysis of the cell cycle, representative histograms for control and curcumin-treated cells are shown. Percentage of cells accumulated in the G1 and G2/M phase of the cell cycle is given on each histogram. **d** Detection of SA-β-gal-activity in control and curcumin-treated cells on the 7th day

VSMCs were treated with 5-7.5 µM curcumin for 7 days and analysis of selected proteins was conducted within the first 24 h and during the following 7 days. We observed quite rapid (several hours after curcumin treatment) activation of the p53/p21 signaling pathway and a decrease in Rb. The latter protein almost completely disappeared after 7 days of treatment (Fig. 1a, b). Cell cycle arrest in the G1 and G2 phase was observed already 24 h after treatment (Fig. 1c) and lasted for several days. The activity of senescence associated-β-galactosidase (SA-β-gal) increased already on day 3 and after 7 days almost all cells were SA- β -gal positive (Fig. 1d). On the basis of this observation we distinguished the initiation phase of cell response to curcumin treatment (up to 24 h) and the execution phase (from 1st to 7th day of treatment) during which the establishment of the senescence phenotype was taking place. Thus, to find out molecular targets of the pro-senescence action of curcumin, we decided to analyze protein expression within the first 24 h.

### Impact of curcumin on the activity of signaling pathways involved in senescence induction

**Fig. 2:**
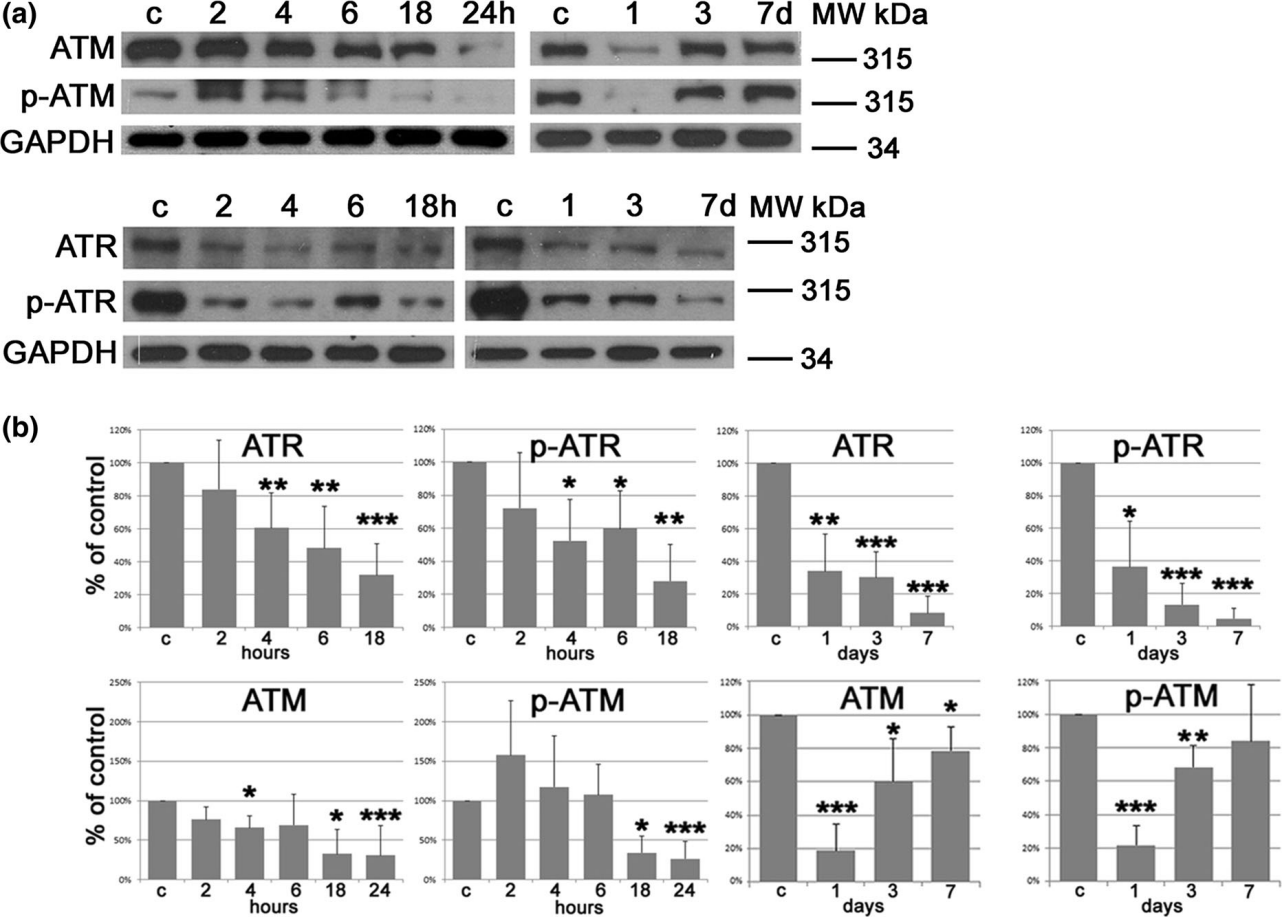
Analysis of proteins involved in cellular senescence and characteristic for selected signaling pathways. **a** Western blot analysis of ATM, p-ATM, ATR, p-ATR. GAPDH served as a loading control. Western blots representative for at least three independent experiments are shown. c—control, untreated cells; **b** Densitometric analysis of proteins analyzed by WB. *p < 0.05, **p < 0.01, ***p < 0.001

Previously we have shown that the activity of ATM in VSMCs treated with curcumin was upregulated after 2 days of treatment (Grabowska et al. 2015). This was not associated with DNA damage suggesting that ATM activation was DNA damage-independent. Moreover, silencing of *ATM* did not reduce the number of senescent cells. Now, we show that after transient elevation (2 h), the level of ATM phosphorylated on Ser1981 started to decrease 6 h after treatment and was almost undetectable after 24 h, after which it began to rise. A significant reduction of ATR level (total and phosphorylated on Ser428) was observed already during the first 24 h and it remained low until the 7th day. Our results suggest that neither ATM nor ATR are involved in the induction or in the execution phase of curcumin-induced senescence (Fig. 2a, b).

As it has been shown that p38 can be the culprit of cell senescence independently of DNA damage response (Iwasa et al. 2003) and we earlier observed increased level of this protein during a 7-days VSMCs treatment with curcumin (Grabowska et al. 2015), we have now checked the level of p38 within a short time period. However, the level of total and active form of p38 protein (phosphorylated on Thr180/Tyr182) remained unchanged during the first 18 h of treatment (not shown). Then, a transient reduction was observed after 24 h (see the results presented on Fig. 5a), followed by a gradual elevation in the subsequent days.

**Fig. 3:**
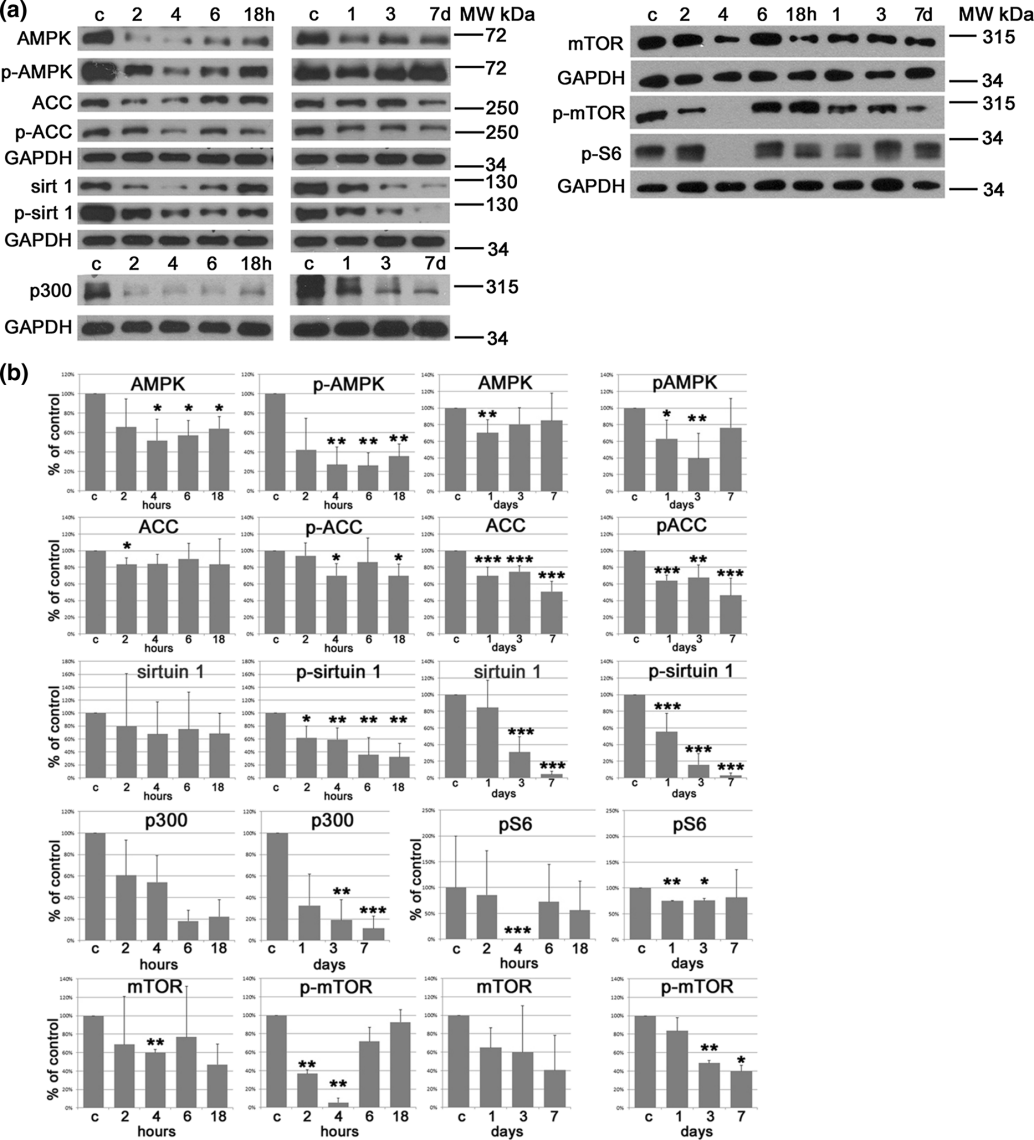
Analysis of proteins involved in cellular senescence and characteristic for selected signaling pathways. **a** Western blot analysis of AMPK, p-AMPK, ACC, p-ACC, sirtuin 1, p-sirtuin 1, p300, mTOR p-mTOR, p-S6. GAPDH served as a loading control. The representative WB for at least three independent experiments are shown. c—control, **b** Densitometric analysis of proteins analyzed by WB. *p < 0.05, **p < 0.01, ***p < 0.001

The level of AMPK, both total and active form (phosphorylated on Thr172), and of its target protein, ACC (total and phosphorylated on Ser79), was first downregulated but, starting from 18 h of treatment, began to rise without, however, reaching the level observed in control cells even after 7-days of treatment with curcumin (Fig. 3a, b). A significant reduction in sirtuin 1 level and activity was also observed in a short and long time period. Moreover, downregulation of p300 level was detected. This enzyme is an acetyltransferase and its activity frequently counteracts the deacetylase activity of sirtuin 1. Surprisingly, we observed a decrease in mTOR level and activity (no signal was consistently observed at the 4 h time point). It was suggested that mTOR was necessary for geroconversion of cell cycle arrested cells (Blagosklonny 2008, 2012). However, mTOR seems not to play such a role in senescence induced by curcumin.

**Fig. 4:**
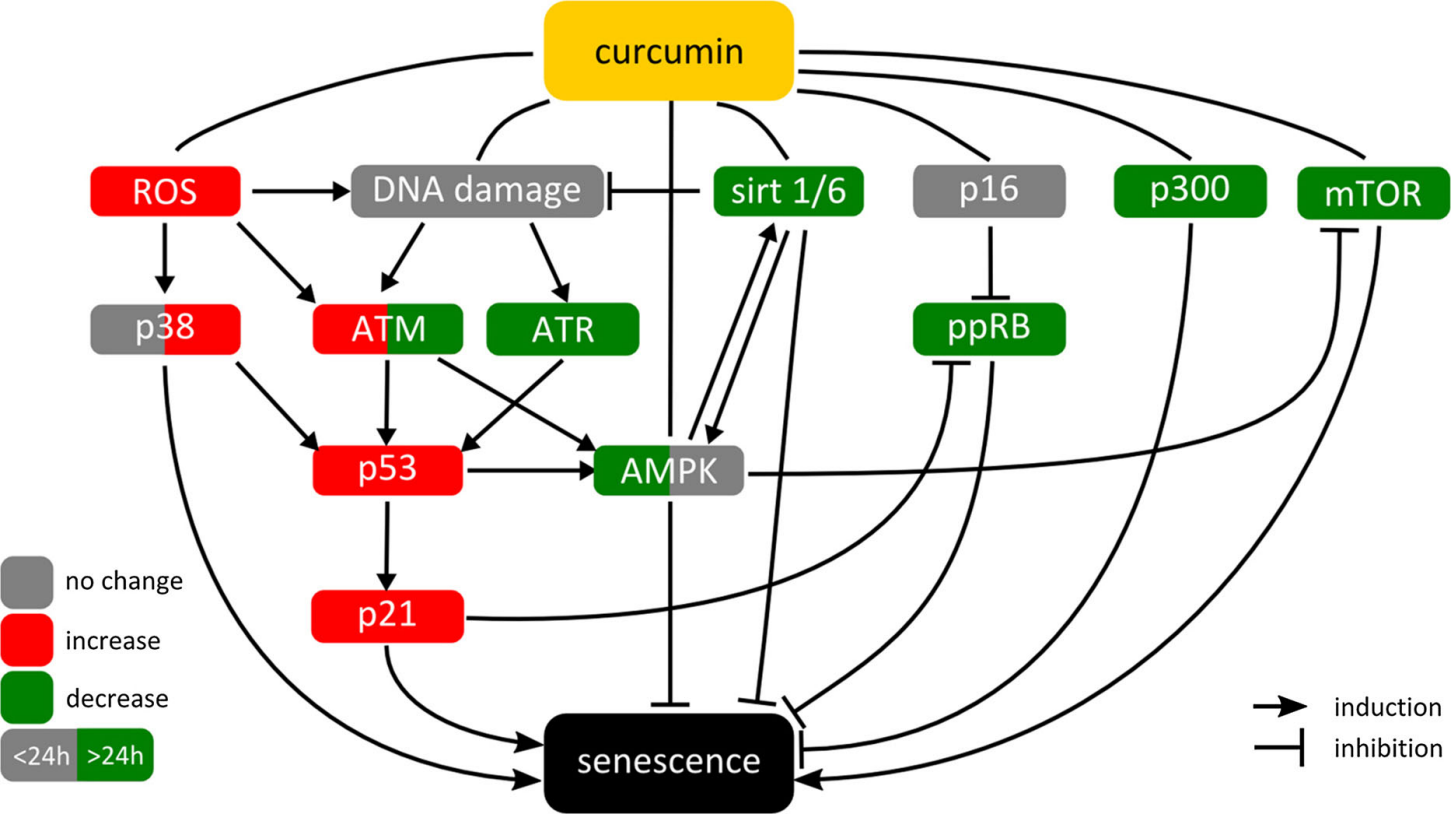
Summary of the pleiotropic activity of curcumin during induction of senescence in VSMCs. Impact of curcumin on different proteins, DNA damage, ROS production, and the interplay between the analyzed proteins/factors

The impact of curcumin on the studied proteins and the interplay between them is depicted in Fig. 4.

### Inhibition of p38 only slightly decreased the number of senescent cells

**Fig. 5:**
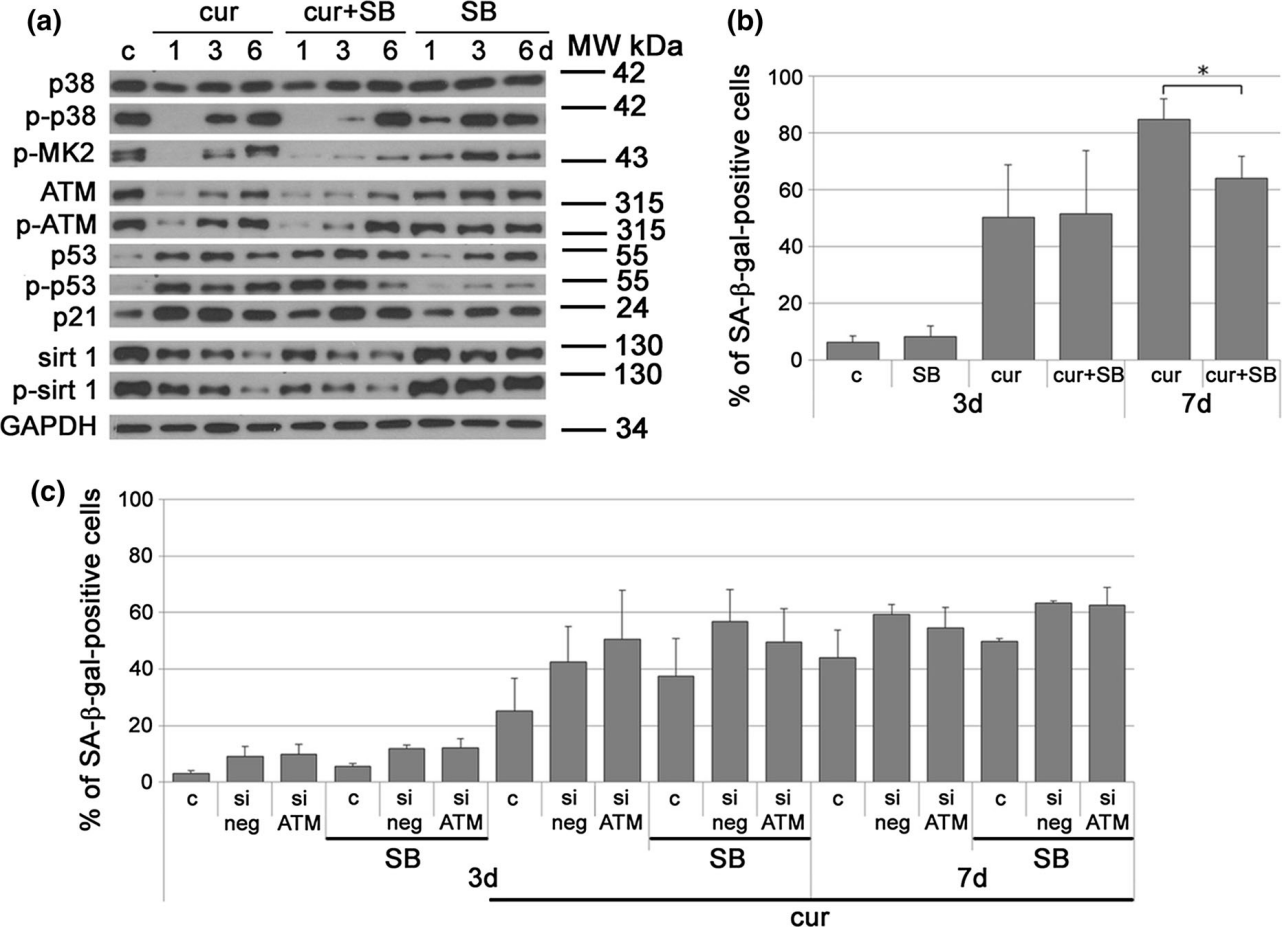
Analysis of the impact of p38 inhibition or p38 inhibition together with ATM silencing on cell senescence progression. **a** Western blot analysis of selected proteins characteristic for the examined signaling pathways involved in senescence of VSMCs. Cells were treated with curcumin (7,5 µM) or curcumin together with p38 inhibitor or with the inhibitor alone. VSMCs were collected after 1, 3, or 6 days. GAPDH served as a loading control. Western blots representative for at least 3 independent experiments are shown. **b** SA-β-gal activity in VSMCs cultured in the presence of curcumin (7,5 µM) or curcumin and p38 inhibitor. Cells were analyzed after 1, 3 and 7 days of treatment. The graph demonstrates the percentage of SA-β-gal-positive cells. **c** SA-β-gal activity in VSMCs cultured with curcumin (7,5 µM) or curcumin together with p38 inhibitor, or in VSMCs with silenced ATM treated with curcumin together with inhibitor. Cells were analyzed after 1, 3 and 6 days of treatment. The graph demonstrates the percentage of SA-β-gal-positive cells. c—control cells, cur—curcumin, SB—p38 inhibitor. p < 0.05, **p < 0.01, ***p < 0.001

Our previous study suggested that p38 could be potentially involved in curcumin-induced senescence, as its level increased consecutively during the 7-days treatment (Grabowska et al. 2015). Now, we have shown that the level of p38 did not change during the first 18 h, which suggest that p38 does not play any role in the initial phase of senescence. Nonetheless, we checked if a subsequent increase in p38 level/activity could be the key element in senescence execution. To analyze this issue we have used SB203580, a p38 inhibitor (hereinafter referred to as SB). SB did not influence the level of p38 but had an impact on its activity since p38 phosphorylation decreased with time and was almost undetectable after 24 h (Fig. 5a). The efficiency of the inhibitor was also demonstrated by analyzing the substrate of p38, namely MK2, phosphorylated on Thr334. The inhibitor alone did not influence cell proliferation (not shown) or the expression of selected proteins involved in senescence progression (Fig. 5a), even though an increased level of total p53 (but not phosphorylated) and a slightly increased level of p21 were observed after 3 days. This could be the effect of overgrowth of proliferating cells, which caused cell cycle arrest. Despite that, after 24 h of curcumin treatment, the level of phosphorylated p38 was undetectable but it increased gradually during the following days. SB, applied together with curcumin, was more effective as partial inhibition of p38 could be observed even after 3 days.

Next, we analyzed the percentage of senescent cells after the double-treatment (curcumin and SB) by studying the activity of SA-β-gal (Fig. 5b). Control cells treated with SB proliferated similarly to cells cultured without SB. We showed that inhibition of p38 activity caused a decrease in the number of senescent cells after a 7-days treatment, in comparison to cells treated with curcumin only. Despite the statistical significance of this result we do not claim that p38 is crucial for curcumin-induced senescence because the inhibitory effect was slight and probably of no biological relevance. Such assumption is justified by the fact that the levels of: p53, ATM/p-ATM, p21 and sirtuin 1 were similar in curcumin and curcumin + SB treated cells (Fig. 5a).

### Simultaneous silencing of ATM and inhibition of p38 did not reduce the number of senescent cells

Because our results showing changes in the level/activity of selected proteins suggested that curcumin-induced senescence is elicited due to activation of at least several signaling pathways and that inhibition of any of them could probably be compensated by activation of another one, we decided to simultaneously inhibit two important pathways, i.e. those engaging p38 and ATM, to elucidate their complementary roles. Our earlier results showed that silencing of ATM alone did not decrease the number of senescent cells (Grabowska et al. 2015). For ATM silencing we used siRNA, which proved very effective in an earlier study (Grabowska et al. 2015). Surprisingly, simultaneous inhibition of p38 and silencing of ATM did not reduce the number of senescent cells in comparison to cells with functional/active proteins (Fig. 5c). To the contrary, the number of senescent cells was slightly higher in ATM-depleted SB-treated cell populations. Our results suggested that curcumin was able to successfully induce senescence of VSMCs even upon inhibition of two major senescence signaling pathways.

### Downregulation of p300 is characteristic for stress-induced senescence of VSMCs

**Fig. 6:**
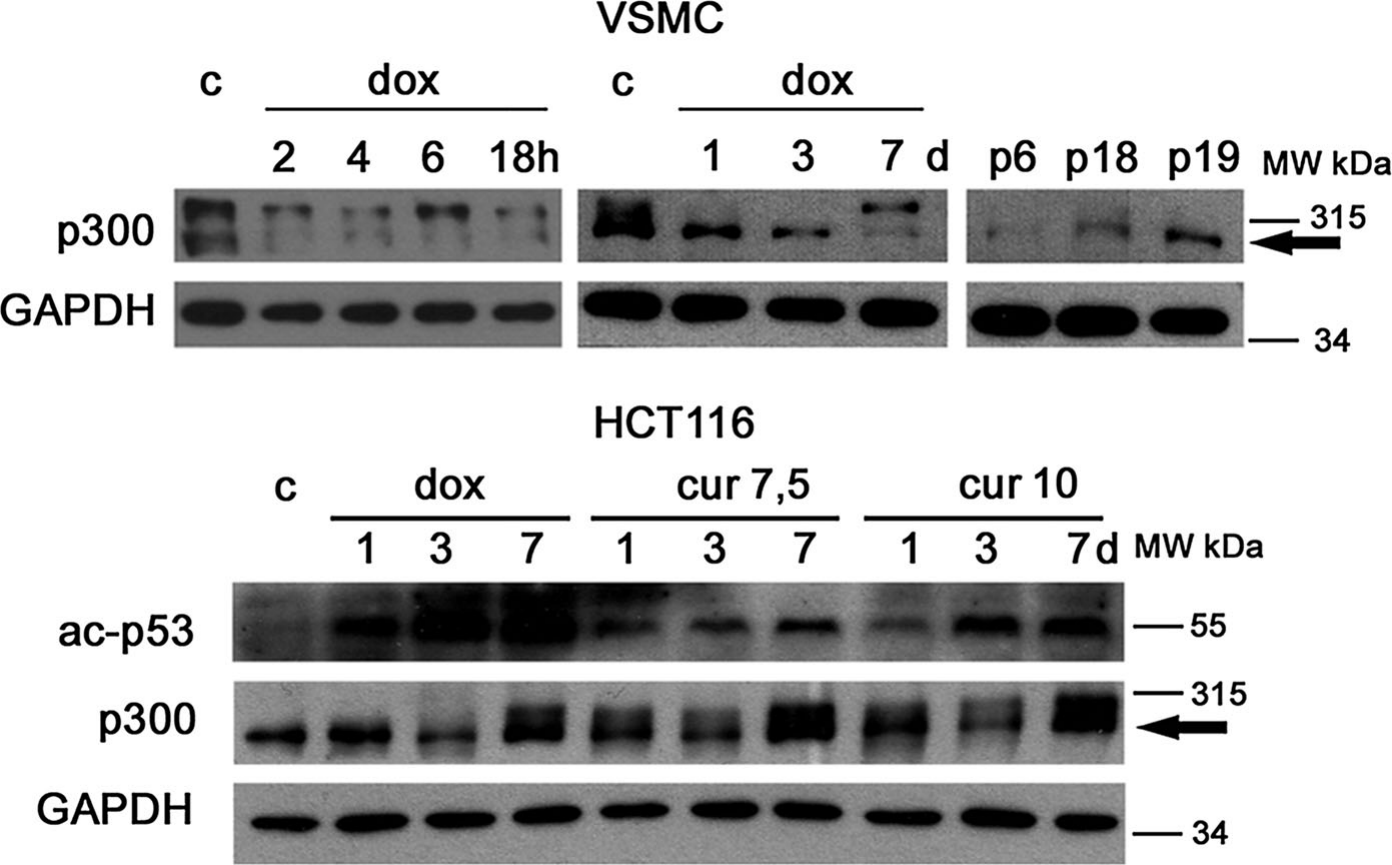
The level of p300 in VSMCs and HCT116 cells induced to senesce by different stimuli. Western blot analysis of p300 in VSMCs induced to senesce by doxorubicin treatment or undergoing replicative senescence (upper panel) and in HCT116 cells after doxorubicin and curcumin treatment. Additionally the level of acetylated p53 (ac-p53) is shown (lower panel). Cur—curcumin, dox—doxorubicin

As p300 downregulation was one of the most spectacular effects of the action of curcumin, we verified if p300 downregulation was specific for curcumin-induced senescence. To this end we compared the level of p300 protein in VSMCs induced to senesce upon curcumin or doxorubicin treatment [doxorubicin-induced senescence of VSMC has been already described by us (Bielak-Zmijewska et al. 2014)]. Analysis of the p300 level (Fig. 6) was also performed for HTC116 tumor cells, for which both models of senescence (following 7,5 and 10 µM curcumin or 100 nM doxorubicin treatment) have already been described (Mosieniak et al. 2012; Sliwinska et al. 2009; Mosieniak et al. 2016). Additionally, we analyzed p300 expression in VSMCs undergoing replicative senescence. In doxorubicin-induced senescence of VSMCs (100 nM doxorubicin for 7 days) the level of p300 diminished already during the first 24 h, and remained low for the following 7 days. However, in replicative senescence, an increase in p300 level was observed. This could indicate that downregulation of p300 is specific for stress induced senescence and not only for curcumin-induced senescence. However, in HCT116 cells, an increased level of p300 was observed both in curcumin- and doxorubicin-induced senescence, which suggests that p300 upregulation is associated with stress induced senescence of this type of cells. Additionally, upregulation of p300 in HCT116 cells was associated with increased level of acetylated p53, which is a target of this enzyme. In VSMCs the level of acetylated p53 was undetectable even in cells senesced in the replicative manner (not shown). The very low level of p300 in VSMCs, which senesced as a result of curcumin treatment, could be due to the possible inhibitory effect of curcumin on HATs. However, such activity was not observed in HCT116 cells.

### In senescent cells there is an increase in the activity of β-glucuronidase

**Fig. 7:**
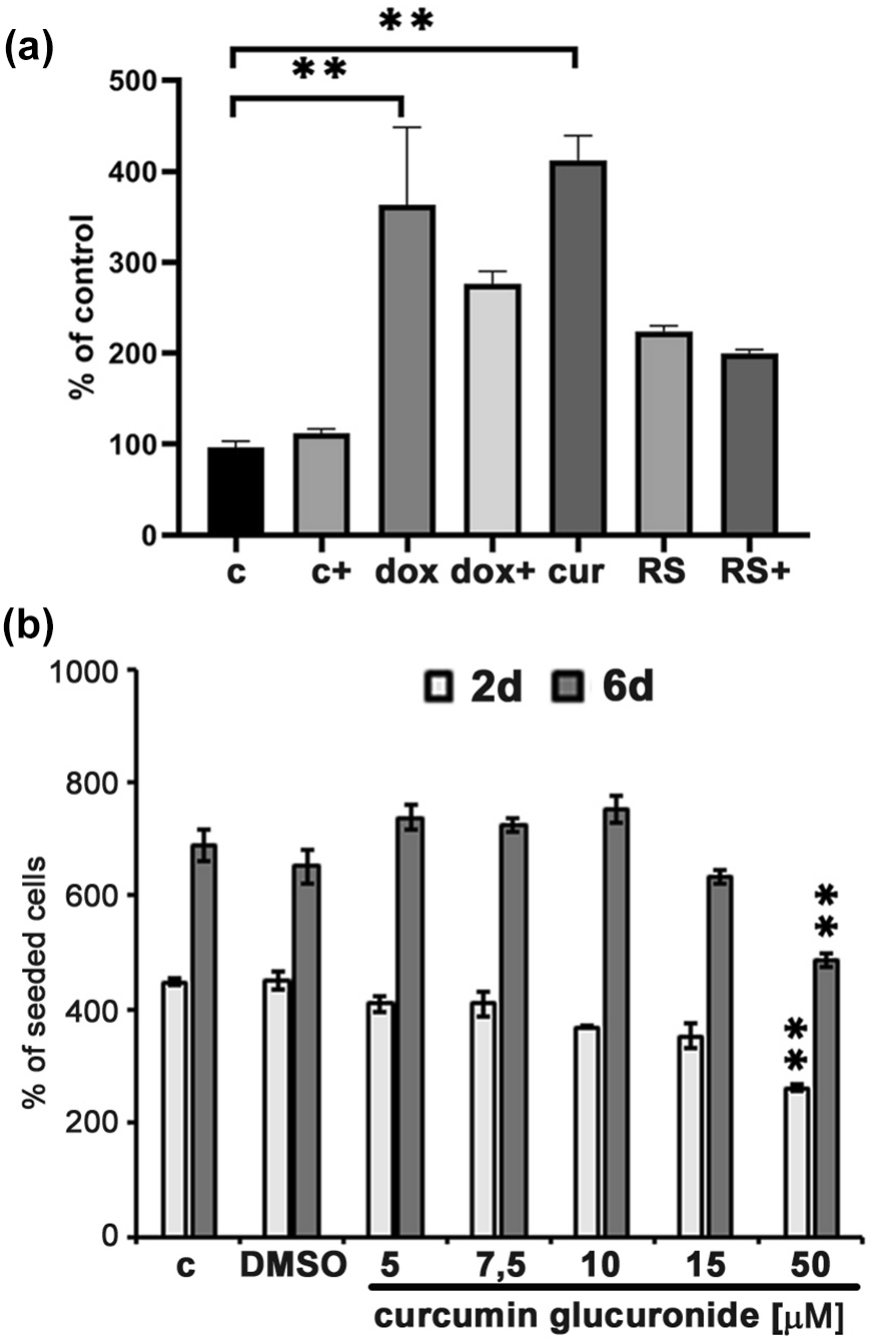
The activity of β-glucuronidase senescent VSMC. **a** Activity of β-glucuronidase was measured in control young cells (c), in young cells treated for 24 h with low, 1 µM, dose of curcumin (c +), cells senesced as a result of 7-days doxorubicin treatment (dox), cells senesced as a result of 7-days doxorubicin treatment and incubated for 24 h with 1 µM curcumin (dox +), cells senesced as a result of 7-days treatment with 7,5 µM curcumin (cur), replicatively senescent cells (RS), replicatively senescent cells, which were additionally incubated for 24 h with 1 µM curcumin (RS +). **b** The impact of curcumin glucuronide on cell proliferation. Cells were treated with curcumin glucuronide in concentrations between 5 and 50 µM. Cells were counted after 2 and 6 days. The graph demonstrates the percentage of cells after 2 or 6-days treatment in comparison to seeded cells. DMSO concentration corresponds to 50 µM curcumin

Curcumin in the organism is metabolized to curcumin glucuronides (mono- or diglucuronides) and such conjugates are much less active than pure curcumin (Vareed et al. 2008; Szymusiak et al. 2016). This can suggest that the pro-senescent effect of a cytostatic concentration of curcumin might not be observed in the organism. On the other hand, there are data showing that inflammation leads to increased activity of the enzyme responsible for deconjugation of curcumin, namely β-glucuronidase. Therefore, we decided to analyze the activity of this enzyme in VSMCs undergoing senescence as a result of both telomere shortening and drug treatment (curcumin or doxorubicin). Moreover, we have checked if curcumin in low concentration, applied for 24 h, leads to increased activity of β-glucuronidase, and if such a dose of curcumin has any impact on the activity of this enzyme in already senescent cells. Our results clearly showed that, in senescent cells, the activity of β-glucuronidase increased (Fig. 7a). This was much more pronounced in prematurely senescent cells than in replicatively senescent ones. Low dose of curcumin (1 µM concentration during 24 h) had no impact on the activity of this enzyme in young cells but slightly decreased its activity in senescent ones. Elevated activity of β-glucuronidase was also observed in cells isolated from human atherosclerotic plaques (not shown), which supports our data.

### Curcumin glucuronides do not induce senescence of VSMC

We also endeavored to analyze if curcumin glucuronide induced similar effects as free curcumin namely, inhibited proliferation and induced senescence. We synthesized curcumin mono-glucuronide, and treated cells at the early passage (young cells) with different concentrations (1–50 µM) of the conjugates for 2 or 6 days. We have shown that conjugates are less active than free curcumin and curcumin glucuronides did not reduce proliferation of VSMCs (Fig. 7b). Some effect of cell division inhibition was observed at very high doses, exceeding 15 µM.

## Discussion

In our attempt to elucidate the mechanism of curcumin-induced senescence, we previously excluded DNA damage or ROS and ATM as the primary cause of senescence (Grabowska et al. 2015). In this study we expanded the analysis to include more detailed time courses and new proteins involved in the senescence process. We showed that neither p38 inhibition alone nor together with ATM silencing spectacularly reduced the number of senescent cells.

Thus, we propose that the induction and execution of senescence by curcumin involves complex interactions between different signaling pathways. A decrease in protein level of: AMPK, sirtuin 1, ATR, p300 and ATM was detected as early as within the first 24 h.

AMPK is a crucial regulator of energy metabolism, both at the cellular and organismal level (Salminen and Kaarniranta 2012). We observed that the level of both total and phosphorylated (active) AMPK decreased during the first 24 h of curcumin treatment and afterwards remained stable but lower than in control. Our earlier results, obtained on VSMCs using a low dose of curcumin (1 µM), which did not affect proliferation, revealed that curcumin upregulated AMPK (Grabowska et al. 2016). The elevation of AMPK level was associated with increased level of sirtuins. Our results strongly suggest that the impact of curcumin on AMPK in VSMCs depends on its concentration. A Similar effect was observed also for sirtuin 1 and 6 (discussed in Bielak-Zmijewska et al. 2019). This could be explained by the well-known biphasic dose response to curcumin, which is convergent with its hormetic properties. Hormesis is related to the induction of stress followed by activation of a protective mechanism involving stress response pathways (discussed in the reviews of Calabrese et al. 2019 and Bielak-Zmijewska et al. 2019). Such a biphasic response was shown for the regulation of many cellular processes (Calabrese et al. 2019). Downregulation of AMPK is associated with senescence and upregulation could have an opposite, protective function (e.g. stimulation of sirtuin 1). There are confusing data concerning the role of AMPK in senescence. Both protecting and inducing effects with regards to senescence, aging and age-related diseases have been reported. Downregulation of AMPK promoted skeletal muscle functional decline with age, which could be explained by an increased number of DNA DSB (double strand breaks) that led to constitutive activation of DNA-PK (Park et al. 2017). Acute AMPK activation protected keratinocytes from H_2_O_2_-induced premature senescence while knockdown of AMPK was sufficient for senescence induction (Ido et al. 2012). On the other hand, increased expression of AMPK led to premature senescence of mouse embryonic and human fibroblasts (Jones et al. 2005; Wang et al. 2003). In the case of VSMCs, there are reports showing that AMPK can inhibit (Lee et al. 2016) or stimulate senescence (Sung et al. 2011). Our results suggest that AMPK negatively regulates senescence in VSMCs. The involvement of AMPK in senescence could be explained, among others, by its ability to downregulate mTOR signaling via inhibition of S6K (Salminen and Kaarniranta 2012). However, we have shown that in curcumin-treated VSMCs both AMPK and mTOR were downregulated. Reduction in mTOR could be caused by curcumin, for which such ability has already been documented (Zhu et al. 2016; Jiao et al. 2016; Sikora et al. 2010). It is possible that these two phenomena, downregulation of AMPK and mTOR, arise independently.

Since the level of AMPK and sirtuin 1 decreased already after 2 h, we suppose that these two proteins could play an important role in senescence initiation. A subsequent decrease in ATM (ATM is, among others, responsible for AMPK stimulation), could additionally downregulate AMPK level. During the following days, the level of ATM gradually increased and, therefore, the AMPK level stabilized. One of the possible mechanisms suppressing AMPK signaling in vivo is low-grade inflammation observed in aging tissues (Viollet et al. 2010) and, in vitro, the secretion of mediators of inflammation produced by senescent cells. Such explanation, however, is not convincing enough for changes observed after 2 h. A second possibility could be that curcumin reduces sirtuin 1 level, which leads to LKB1 decrease, the consequence of which could be AMPK reduction. In turn, lower level/activity of AMPK could be responsible for diminished activity of sirtuin 1. The molecular bases of this relationship have already been described (reviewed in Grabowska et al. 2017). It has been shown that reduction in the level or activity of sirtuin 1 can lead to premature senescence of endothelial cells (Ota et al. 2007; Menghini et al. 2009). Sirtuin downregulation could be induced by acute or prolonged oxidative conditions (Santos et al. 2016) and, as our earlier results show, curcumin increases ROS production (Grabowska et al. 2015). Attenuation of sirtuin 1 deacetylase activity enhances p53 acetylation and its stabilization leading to premature senescence (Tran et al. 2014). However, we were not able to detect ac-p53 in VSMCs, contrary to HCT cells, where the ac-p53 level increased after both curcumin and doxorubicin treatment. Lack of ac-p53 in VSMCs could be the consequence of p300 depletion. Downregulation of p300 could be, per se, responsible for curcumin-induced senescence (Prieur et al. 2011). Its level decreased already after 2 h of curcumin treatment and remained low until the 7th day. Curcumin is a HAT inhibitor and fibroblasts with reduced p300 HAT activity underwent senescence manifested by G2/M cell cycle arrest but not accompanied by DNA damage, similarly to what was observed in VSMCs (Grabowska et al. 2015). However, senescence of fibroblasts was p53-, p21- and p16-independent and was evoked by hypoacetylation of H3 and H4. Due to the pleiotropic action of curcumin we cannot consider it as a specific inhibitor of p300. The level of p300 could also be reduced due to its increased proteosomal degradation promoted by curcumin, as has been shown by Marcu et al. (Marcu et al. 2006). The role of p300 in cellular senescence is not clear. However, changes in chromatin organization could be involved in curcumin-induced senescence. Chromatin remodeling does not induce DNA damage but leads to activation of ATM (Bakkenist and Kastan 2003, 2015; Kaidi and Jackson 2013). ATM is the initiating kinase in DNA damage response and we observed its activation in VSMCs despite the lack of DNA lesions (Grabowska et al. 2015). It has been shown that ATM could be activated by treatment with histone deacetylase inhibitors (Bakkenist and Kastan 2003, 2015) and curcumin was shown to inhibit deacetylases as well.

The ability of curcumin to induce senescence must be taken into account if prophylactic and therapeutic application of this compound is considered. Elucidation of the mechanism of curcumin-induced senescence may be helpful in predicting when such ability could be beneficial and when detrimental for cells building the vascular system. There are, in general, two faces of cellular senescence: it is indispensable for regeneration of tissues and limits spreading of cancer cells but, on the other hand, it is associated with aging and age-related diseases. There are some suggestions that curcumin can induce “beneficial senescence” in cancer cells or in CAF, because it stops proliferation or reduces the metastatic potential of cancer cells (Mosieniak et al. 2012; Hendrayani et al. 2013; Mosieniak et al. 2016). Moreover, by promoting senescence in activated HSC, curcumin protected the tissue from fibrosis (Jin et al. 2017). Similarly to VSMCs, both CAF and HSC senesced without DNA lesions. There is an opinion that senescence that does not involve DNA injury could be considered as beneficial. In the context of atherosclerosis, it has been suggested that at a certain stage of disease progression, senescence fulfills a protective role (Muñoz-Espín and Serrano 2014). However, it cannot be excluded that curcumin is able to impair the functionality of tissues and organs by inducing senescence in normal cells.

Nevertheless, it cannot be ruled out that curcumin-induced senescence does not occur in the organism or occurs only under very specific circumstances. It must be taken into account that dietary curcumin is metabolized and the in vivo effects are a combination of the impact of curcumin and its metabolites. Curcumin is glucuronided and such form is less active than curcumin itself (Vareed et al. 2008; Szymusiak et al. 2016) similarly to other formulations of this compound e.g. nano-curcumin. Our data also indicate that curcumin glucuronides did not impair proliferation of VSMC at early passages. Some reduction of cell division was observed but at non-physiological doses (15, 50 µM). The lack of the impact of the conjugates on the division of cancer cells has been shown using similar or higher doses (Pal et al. 2014). An increased level of curcumin in the plasma was observed following pre-treatment with a lysosomal enzyme, β-glucuronidase, and was interpreted as a result of hydrolysis of the glucuronide moiety (Takahashi et al. 2009; Sasaki et al. 2011; Kanai et al. 2012). Similar effect was already observed for another polyphenol, quercetin (Perez et al. 2014; Menendez et al. 2011). Our results indicated that the activity of β-glucuronidase increased in senescent VSMCs (independently of the manner of senescence induction). The activity of β -glucuronidase increases during inflammation (Peyrol et al. 2018). This enzyme can be released from granulocytes, including neutrophils (Marshall et al. 1988). Upregulated level of mediators of inflammation is associated with both ageing and age-related diseases, including atherosclerosis. Therefore, inflammatory conditions could promote an increase in concentration of free curcumin in the plasma and tissues, e.g. at the site of atherosclerotic plaques (our preliminary results showed an increased activity of β-glucuronidase in cells isolated from atherosclerotic plaques—not shown). Infiltration of macrophages through an impaired endothelial barrier and subsequent accumulation of foam cells in the atherosclerotic plaques play an important role in atherosclerosis. Macrophages secrete β-glucuronidase whose extracellular activity is essential for biological activation of glucuronides (Kawai 2014). On the one hand, curcumin can be released from the conjugates during inflammation, on the other hand, curcumin is able to reduce the level of the mediators of inflammation. The question is which effect dominates. Cytostatic properties of curcumin have already been exploited in reducing stent restenosis, which occurs as a result of angioplasty (curcumin-coated vs. bare metal stents) (Jang et al. 2009); however, cellular senescence has not been studied in this case. Even though curcumin is metabolized to glucuronide and it is documented that conjugates are less active, it does not preclude the effectiveness of curcumin. Conjugates can accumulate in the tissues and it is proposed that glucuronide can act as a prodrug. Such accumulation and subsequent release of the free compound in tumor tissues has been shown for other natural factors, present in ginger extract, which are also metabolized by glucuronisation (Mukkavilli et al. 2018). It has been suggested that application of curcumin monoglucuronide exerts an anti-cancer effect (Ozawa et al. 2017). It cannot be excluded that in the organism also the conjugates can modulate ageing/senescence. In our experiments we have treated young cells, with low activity of β-glucuronidase, in non-inflammatory experimental conditions. In the organism such conjugates could act differently. It is worth to mention that β-glucuronidase is also produced by microbiota (McIntosh et al. 2012). Curcumin is able to modulate the diversity of microbiota (Ohno et al. 2017; Zhang et al. 2017; Shen et al. 2017) and in this manner influence the functioning of the organism. Summarizing, the in vitro obtained results show the ability of curcumin to induce cell senescence. They should, however, be verified in studies on animal models with concern to the age of the animal and its inflammatory status.

## Conclusions

Curcumin, which has been shown to have many beneficial effects, including an anti-aging effect in model organisms, unexpectedly induced senescence of VSMCs. Cellular senescence, depending on the context, could be either beneficial (protection from diseas) or harmful (disease promotion). Indeed, curcumin-induced senescence of cells building the vasculature could either protect them from atherosclerosis or support it. It is difficult to judge definitely, which signaling pathway plays a key role and to establish, which changes in protein expression or activity are primary and which secondary in curcumin-senescent cells. It is possible that many pathways are activated independently because of the pleiotropic activity of curcumin and none of them is the leading one (Fig. 4). Based on our observation, we propose that the induction and maintenance of senescence is regulated by time dependent interplay between a number of key proteins. The ability to induce senescence does not exclude anti-ageing properties. It depends on the concentration of curcumin. The above mentioned biphasic dose response related to curcumin activity can be involved in the modulation of the ageing process. To elucidate this issue a comprehensive in vivo study will be necessary.

## Electronic supplementary material

Below is the link to the electronic supplementary material.

Supplementary material 1 (PDF 1881 kb)
