# Supplementary material for: Curcumin induces multiple signaling pathways leading to vascular smooth muscle cell senescence
Source: Biogerontology. 2019 Aug 1;20(6):783–98. doi: 10.1007/s10522-019-09825-2 (PMC6790191; doi:10.1007/s10522-019-09825-2)
Supplement: Supplementary file 1 — Supplementary material 1 (PDF 1881 kb) [file 10522_2019_9825_MOESM1_ESM.pdf]

Supplementary data
Curcumin-glucuronide synthesis

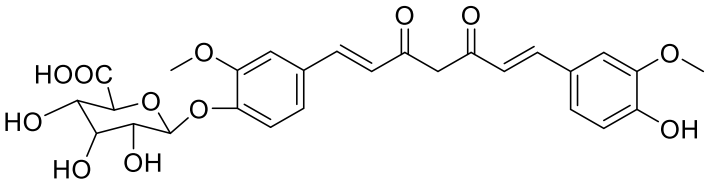

Reagents, Instrumentation 2

Synthesis of Methyl [1-O-(4′-formyl-2′-methoxyphenyl)-2,3,4-tri-O-acetyl-β-d-glucopyranosiduronate) (1) 3

4-(4-hydroxy-3-methoxyphenyl)-3-buten-2-one (2) 3

5-Hydroxy-1-(4-hydroxy-3-methoxyphenyl)-1,4-hexadien-3-one (3)..4

Mono-[methyl 2,3,4-tri-O-acetyl-β-d-glucopyranosiduronate]-curcumin (4) 5

Mono-(β-d-glucopyranosiduronic acid)-curcumin (curcumin monoglucuronide) (5) 6

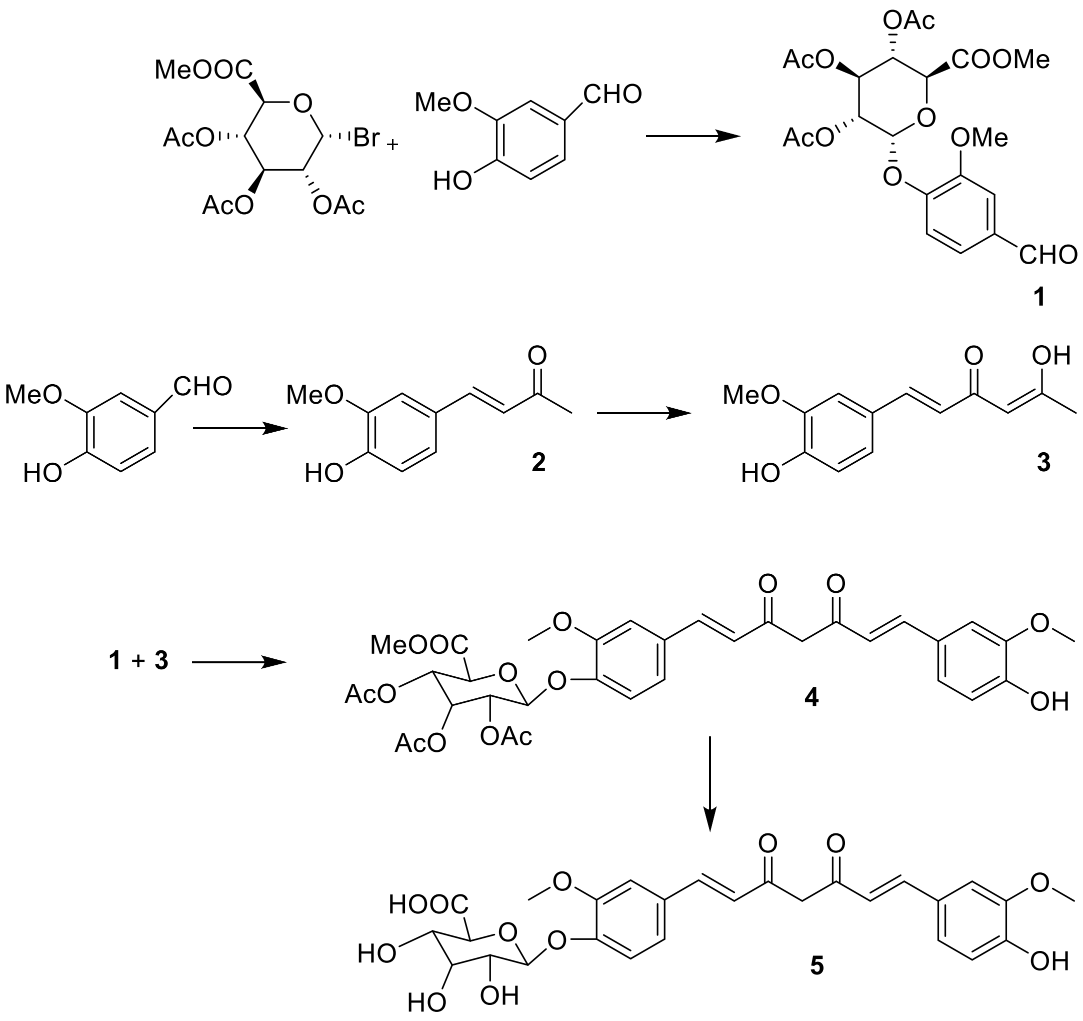

Scheme 1. General synthesis of mono-(β-d-glucopyranosiduronic acid)-curcumin (curcumin monoglucuronide)

Reagents, Instrumentation

All chemicals were purchased from Sigma-Aldrich, Acros Organics and Avantor Performance Materials Poland S.A. Solvents (hexane, acetone, dichloromethane, chloroform, methanol, and ethanol) were used as received, ethyl acetate was dried over potassium carbonate. 99,9% grade N,N-dimethyl formamide (DMF) and tetrahydrofuran (THF) was used as received. ^{1}H and ^{13}C-NMR spectra were recorded on a Varian 300 MHz NMR Instrument (unless otherwise indicated). Chemical shifts were reported in ppm relative to TMS as reference standard. Coupling constants were presented in Hertz and splitting pattern were assigned as s, singlet; d, doublet; t, triplet; m, multiplet, and bs, broad singlet. Mass spectra was recorded on Mas Quattro TOF-LCT with ESI ionization spectrometer. Column chromatography was performed on silica gel (60–120 mesh).

Synthesis of Methyl [1-O-(4′-formyl-2′-methoxyphenyl)-2,3,4-tri-O-acetyl-β-d-glucopyranosiduronate) (1)

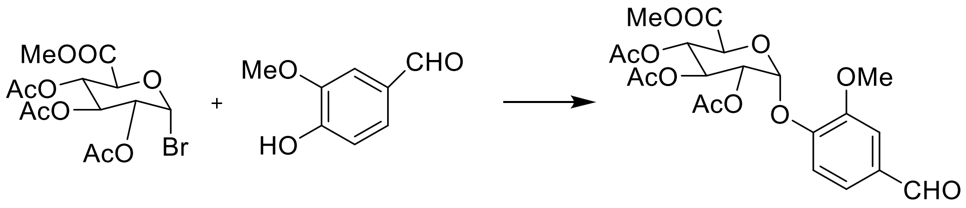

1

Scheme 2. General synthesis of **1**.

Synthesis was performed according to *Antioxidants* **2015**, *4*, 750-767; doi:10.3390/antiox4040750 with some modifications.

Methyl 1-bromo-2,3,4-tri-O-acetyl-α-d-glucopyranosiduronate (0,5 g, 1.26 mmol) and vanillin (0.203 g, 1.34 mmol) were dissolved in 2.5 mL of chloroform and then 2.5 mL of 1M sodium hydroxide solution and tetrabutyl ammonium bromide (0,5 g, 1,34 mmol) were added. The reaction mixture was stirred at 50°C for 6 h. It was cooled to RT and diluted with 5 mL of $CHCl_3$. Organic layer was washed with water (2 mL×2), cold 1M HCl solution (2 mL×3), water (2 mL×2) and brine (2 mL×1), respectively. It was dried over sodium sulfate, filtered, concentrated and purified by column chromatography (silica gel; 1:1 hexane-ethyl acetate) to afforded compound **1** as white powder. Yield 0,2699 g (53%), R_f 0.38 (1:1 hexane-ethyl acetate). ^{1}H NMR ($CDCl_3$): δ 9.90 (s, 1H, CHO), 7.45–7.41 (m, 2H), 7.27 (d, 1H, *J* = 3,5 Hz), 5.31–5.23 (t, 3H), 5.20 (d, 1H, *J* = 7.1 Hz, H-1′), 4.18 (d, 1H, *J* = 9.3 Hz, H-4'), 3.89 (s, 3H, -C6H3OCH$_3$), 3.73 (s, 3H, -COOCH$_3$), 2.08. 2.06. 2.05 (3s, 9H, OCOCH$_3$). ^{13}C-NMR ($CDCl_3$): δ 13C NMR (75 MHz, CDCl3) δ 190.93, 170.07, 169.33, 169.17, 166.79, 151.04, 150.78, 133.00, 125.53, 118.77, 110.68, 99.63, 72.71, 71.52, 70.89, 69.01, 56.09, 52.97, 30.92, 20.62, 20.59, 20.51.

4-(4-hydroxy-3-methoxyphenyl)-3-buten-2-one (2)

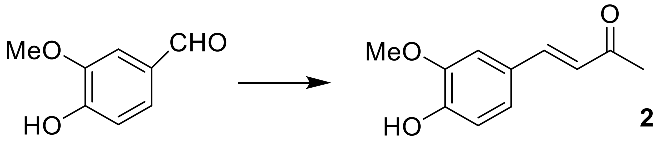

2

Scheme 3. General synthesis of **2**.

Synthesis was performed according to *Antioxidants* **2015**, *4*, 750-767; doi:10.3390/antiox4040750 with some modifications.

Vanillin (10 g, 65.8 mmol) was dissolved in 60 mL of acetone and then a solution of sodium hydroxide (3g, 74 mmol in 40 mL of water) was added. The reaction mixture was stirred at 0–10°C for 1 h. Ice-bath was removed and it was stirred at RT for 48 h. The reaction mixture was evaporated to dryness and acidified with aqueous 1M hydrochloric acid solution. Precipitated solid was filtered, washed with water (50 mL×3) and dissolved in 100 mL of dichloromethane. The solution was dried over sodium sulfate, filtered and evaporated. The crude product was stirred with 50 mL of ethyl acetate for 30 min. Precipitated solid was filtered, washed with ethyl acetate (25 mL×2), and dried under high vacuum to obtain **2** as yellow powder. Yield 9 g (71%). R_f 0.56 (1:1 hexane-ethyl acetate). ^{1}H-NMR ($CDCl_3$): δ 7.45 (d, 1H, *J* = 16.2 Hz, H-4), 7.11–7.06 (m, 2H), 6.93 (d, 1H, *J* = 8.1 Hz), 6.59 (d, 1H, *J* = 16.2 Hz, H-3), 3.93 (s, 3H, $-C_6H_3OCH_3$), 2.37 (s, 3H, H-1). ^{13}C-NMR ($CDCl_3$): δ 198.47, 148.28, 146.89, 143.78, 126.81, 124.98, 123.53, 114.83, 109.33, 55.96, 27.31.

5-Hydroxy-1-(4-hydroxy-3-methoxyphenyl)-1,4-hexadien-3-one (3)

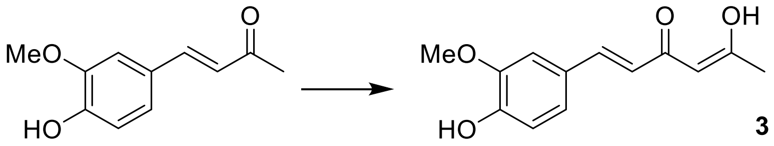

3

Scheme 4. General synthesis of **3**.

Synthesis was performed according to *Antioxidants* **2015**, *4*, 750-767; doi:10.3390/antiox4040750 with some modifications.

To a mixture of dry tetrahydrofuran (3.4 mL) and ethanol (0.4 mL, 7 mmol), sodium metal (0.14 g, 7 mmol) was added and it was refluxed at 95–100°C until all metallic sodium reacted (3-4 h). Then a solution of compound **2** (0.33 g, 1.74 mmol) in tetrahydrofuran (2 mL) and ethyl acetate (2 mL) was added dropwise (~5 min). The reaction mixture was stirred at 95–100°C for 4 h, cooled to RT and diluted with 7 mL of ethyl acetate. Organic layer was washed with cold 1M hydrochloric acid solution (7 mL×3), water (4 mL×1) and brine (4 mL×1), respectively. The crude material was purified by column chromatography (silica gel, 2:1 hexane-ethyl acetate) and the desired fractions were collected, concentrated and dried under high vacuum to afford compound **3** as yellow powder. Yield 0,146 g (45%). R_f 0.61 (2:1 hexane-ethyl acetate). ^{1}H NMR ($CDCl_3$): δ 9.82 (s, 1H), 7.53 (d, 1H, *J* = 15.8 Hz, H-6), 7.01 (d, 1H, *J* = 1.9 Hz), 7.09–7.01 (m, 1H), 6.91 (d, 1H, *J* = 8.2 Hz), 6.32 (d, 1H, *J* = 15.8 Hz, H-5), 5.63 (s, 1H), 3.92 (s, 3H, $-C_6H_3OCH_3$), 2.15 (s, 3H, H-1). ^{13}C NMR ($CDCl_3$): δ 197.02, 177.97, 147.77, 146.84, 140.1, 127.63, 122.66, 120.27, 114.86, 109.54, 100.7, 55.94, 26.83.

Mono-[methyl 2,3,4-tri-O-acetyl-β-d-glucopyranosiduronate]-curcumin (4)

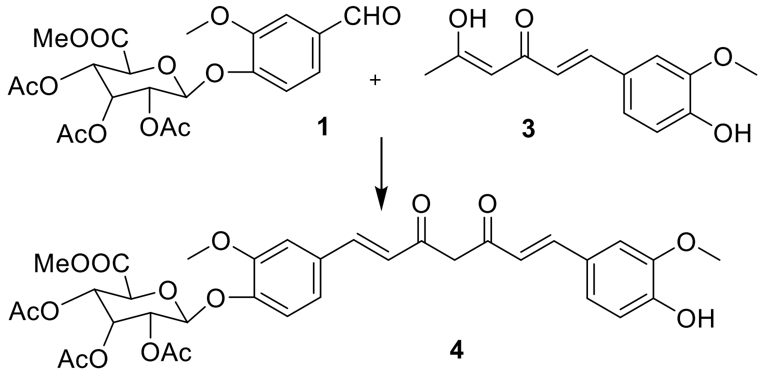

Scheme 5. General synthesis of **4**.

Synthesis was performed according to *Antioxidants* **2015**, *4*, 750-767; doi:10.3390/antiox4040750 with some modifications.

Compound **3** (0.1 g, 0.43 mmol) was dissolved in DMF (3.4 mL) and then boric anhydride (0.05 g 0.68 mmol) of was added. After 30 min of stirring at 75–80°C tri-izopropyl borate (0.7 mL 3 mmol) was added and stirred for additional 1h. A solution of compound **1** (0.2 g, 0.43 mmol) in DMF (3.4 mL) was then added followed by n-butyl amine (0.02 mL 0.17 mmol). The reaction mixture was stirred at 75–80°C for 5 h, cooled to 50–55°C, 10% aqueous acetic acid (6.7 mL) was then added and stirred for 1 h. After cooling to RT, 3 mL of water was added and the mixture was stirred for 30 min. The precipitated solid was separated by a centrifuge (5000RPM), dissolved in 8 mL of ethyl acetate and washed with cold 1M hydrochloric acid solution (3 mL×3), water-brine (4:1) (5 mL×3) and brine (5 mL×1) respectively. Organic layer was dried over sodium sulfate, filtered and evaporated. The crude material was purified by column chromatography (silica gel, 1:1 hexane-ethyl acetate). The desired fractions were collected, concentrated and dried under high vacuum to afford compound **4** as yellow powder. Yield 0,074 g (26%). Rf 0.25 (1:1, hexane:ethyl acetate). ^{1}H NMR ($CDCl_3$): δ 7.62 (d, 1H, *J* = 8.8 Hz), 7.57 (d, 1H, *J* = 8.9 Hz), 7.16–7.05 (m, 5H), 6.94 (d, 1H, *J* = 8.1 Hz), 6.53 (d, 1H, *J* = 8.1 Hz), 6.47 (d, 1H, *J* = 8.0 Hz), 5.82 (s, 1H), 5.36–5.30 (m, 3H), 5.09 (d, 1H, *J* = 6.7 Hz, H-1), 4.12 (d, 1H, *J* = 9.3 Hz, H-4), 3.95 (s, 3H), 3.87 (s, 3H), 3.74 (s, 3H), 2.09, 2.06 and 2.06 (3s, 9H). ^{13}C-NMR ($CDCl_3$): δ 184.16, 182.07, 170.99, 169.97, 169.26, 169.14, 167.63, 166.86, 150.70, 148.19, 147.29, 147.00, 140.97, 139.40, 132.38, 131.78, 130.81, 128.71, 127.38, 123.47, 122.92, 121.48, 119.93, 114.99, 111.60, 109.79, 101.35, 100.14, 72.51, 71.69, 70.97, 69.16, 56.00, 55.83, 52.80, 20.89, 20.50, 20.37.

Mono-(β-d-glucopyranosiduronic acid)-curcumin (curcumin monoglucuronide) (5)

Synthesis was performed according to *Bioorg. Med. Chem.* **2014**, *22*, 435-439 https://doi.org/10.1016/j.bmc.2013.11.006 with some modifications.

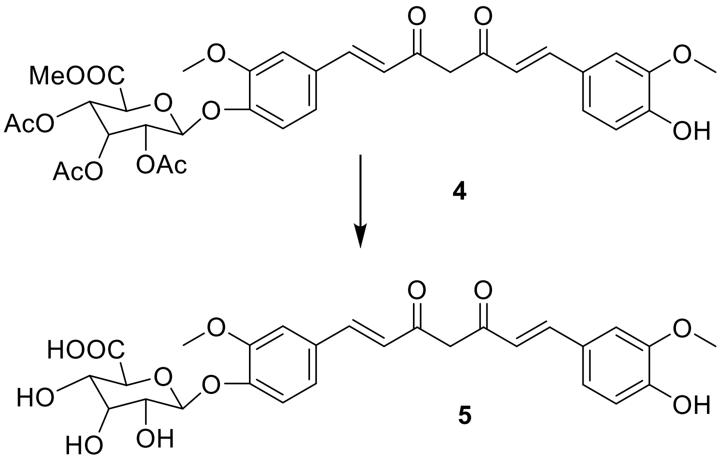

Scheme 6. General synthesis of **5**.

Aqueous 1M NaOH solution (1.4 mL) was added drop wise to a cooled (ice-water bath) solution of compound **4** (0.050 mg, 0.074 mmol) in methanol (1.4 mL). The resulting solution was then stirred for 3h at 0°C and then pH was adjusted to 4 by addition of 50% aqueous formic acid. The yellow solid was separated by a centrifuge (5000RPM) and purified by column chromatography on silica gel eluted with 4:1, 2:1 and 1:1 chloroform–methanol. The fractions were collected, concentrated and dried under vacuum to obtain the desired compound **5** as yellow powder. Yield: 0.28 g (56%). Rf = 0.47 (1:1 $CHCl_3$-MeOH). MP 159°C; ^{1}H NMR (500MHz CD_3OD): δ 9.87 (s, COOH), 7.59 (d, *J* = 13.0 Hz, 2H), 7.27–7.19 (m, 4H), 7.12 (d, *J* = 6.9 Hz, 1H), 6.83 (d, *J* = 8.2 Hz, 1H), 6.71 (d, *J* = 15.9 Hz, 1H), 6.65 (d, *J* = 15.8 Hz, 1H), 6.22 (s, 1H), 6.01 (s, 1H), 4.97 (d, *J* = 7.3 Hz, 1H), 4.58 (s, 1H), 3.92 (s, 6H), 3.76 (d, *J* = 9.2 Hz, 1H), 3.57–3.53 (m, 3H), 3.35 (s, 1H). LRMS (ESI^-) (C27H28O12) calc 544.158 found 543.1 (M^-) and 565.2 (M^-Na).

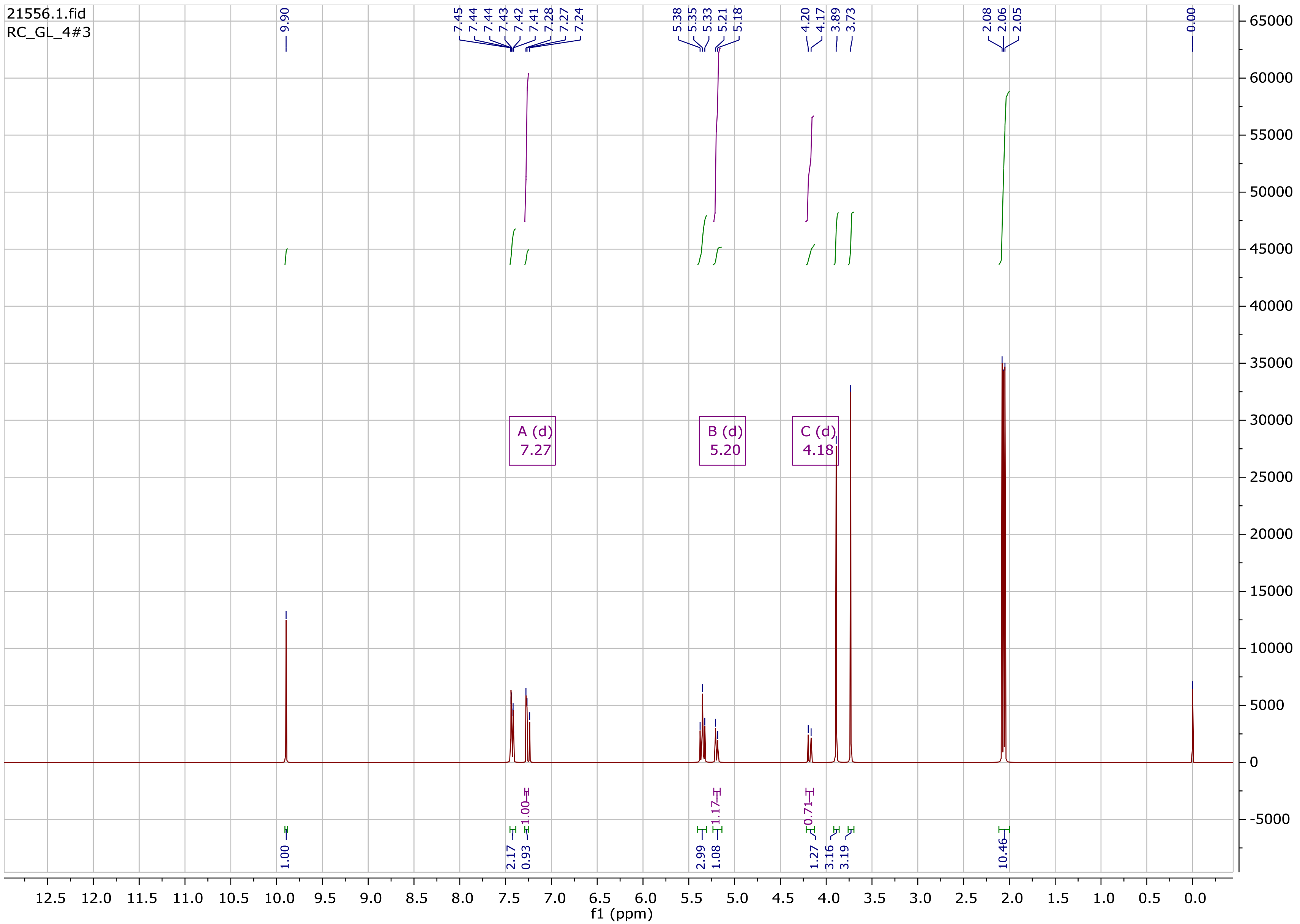

Figure 1. ^{1}H NMR spectrum of **1**.

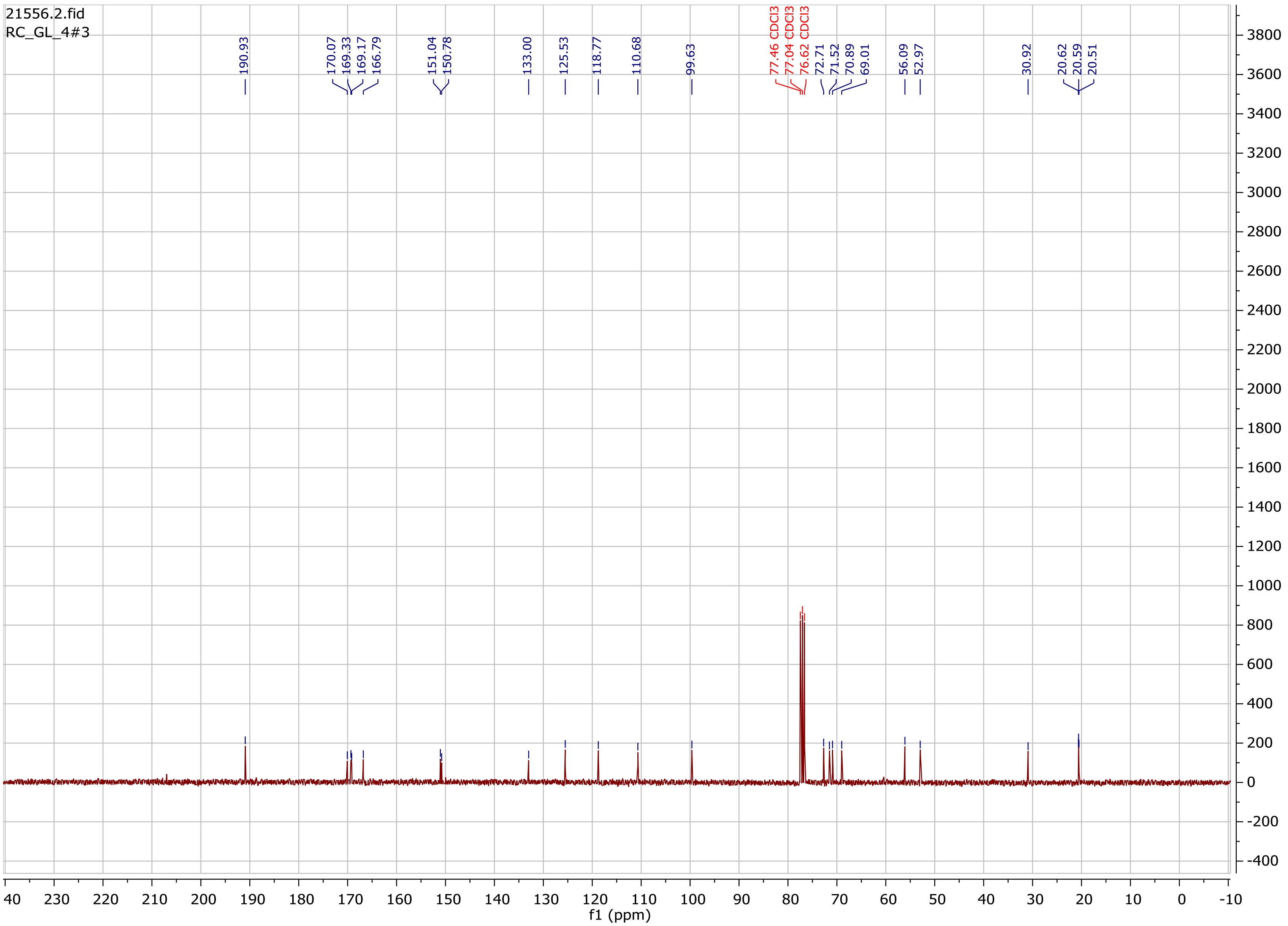

Figure 2. ^{13}C NMR spectrum of **1**.

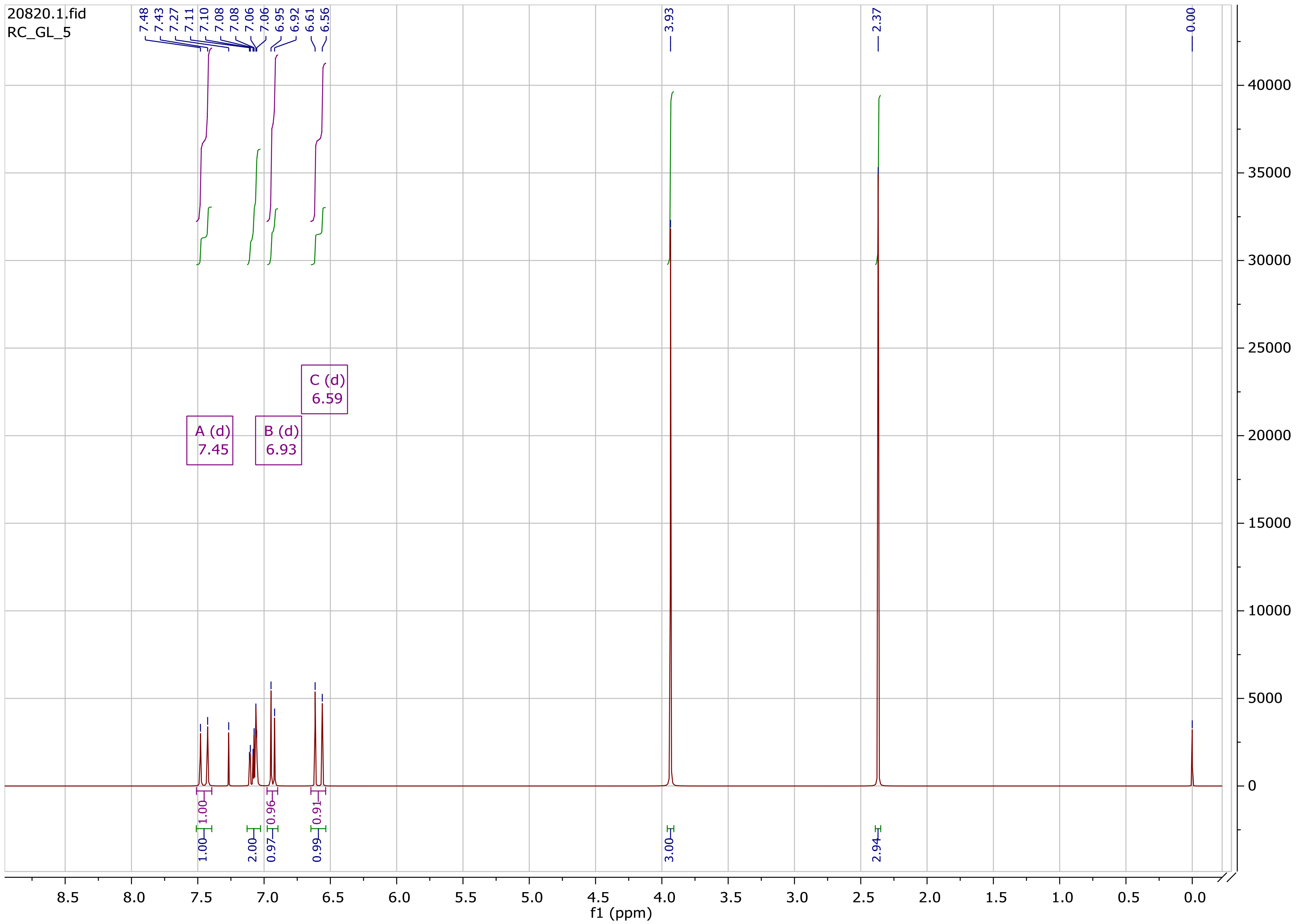

Figure 3. ^{1}H NMR spectrum of **2**.

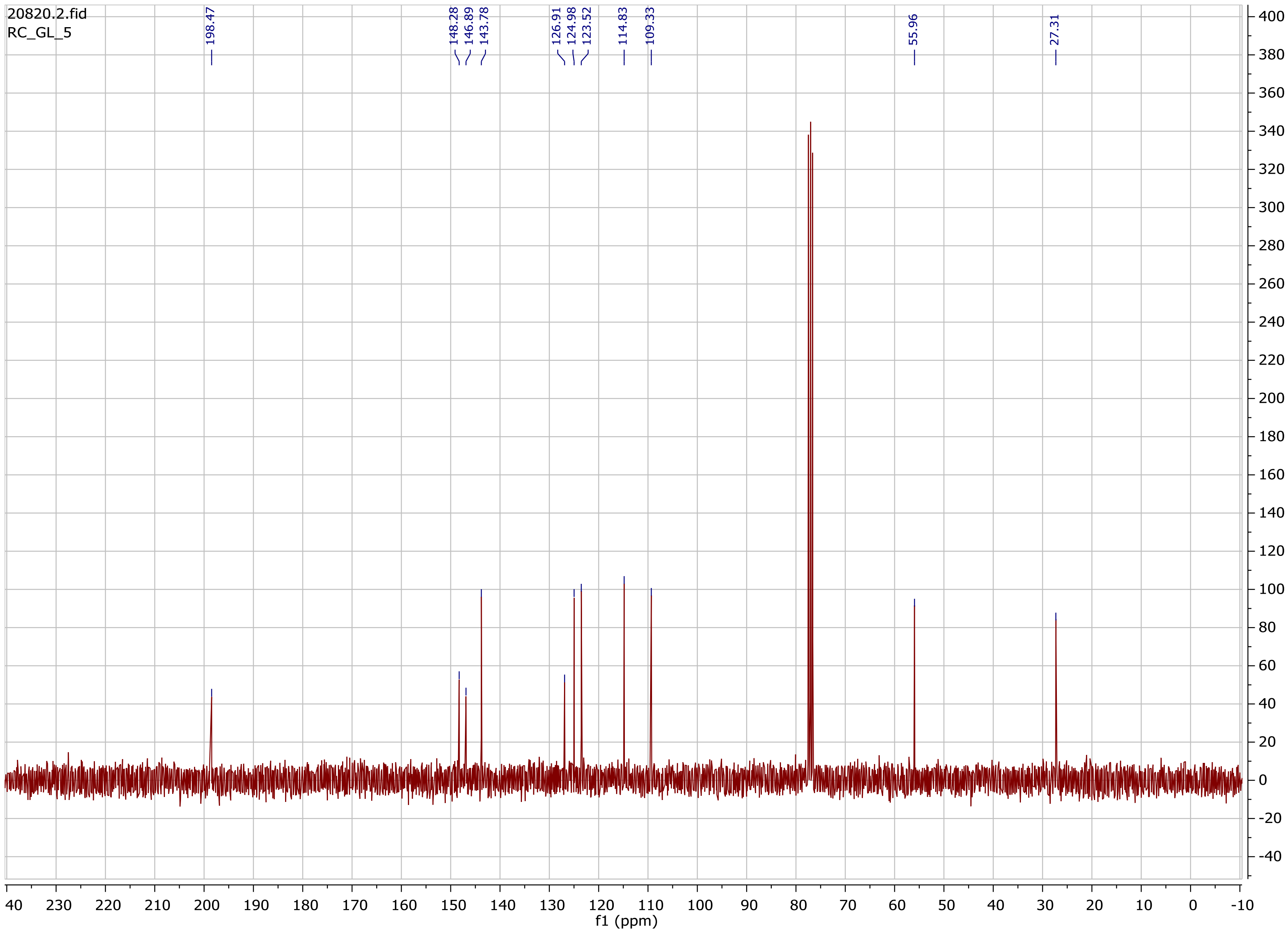

Figure 4. ^{13}C NMR spectrum of **2**

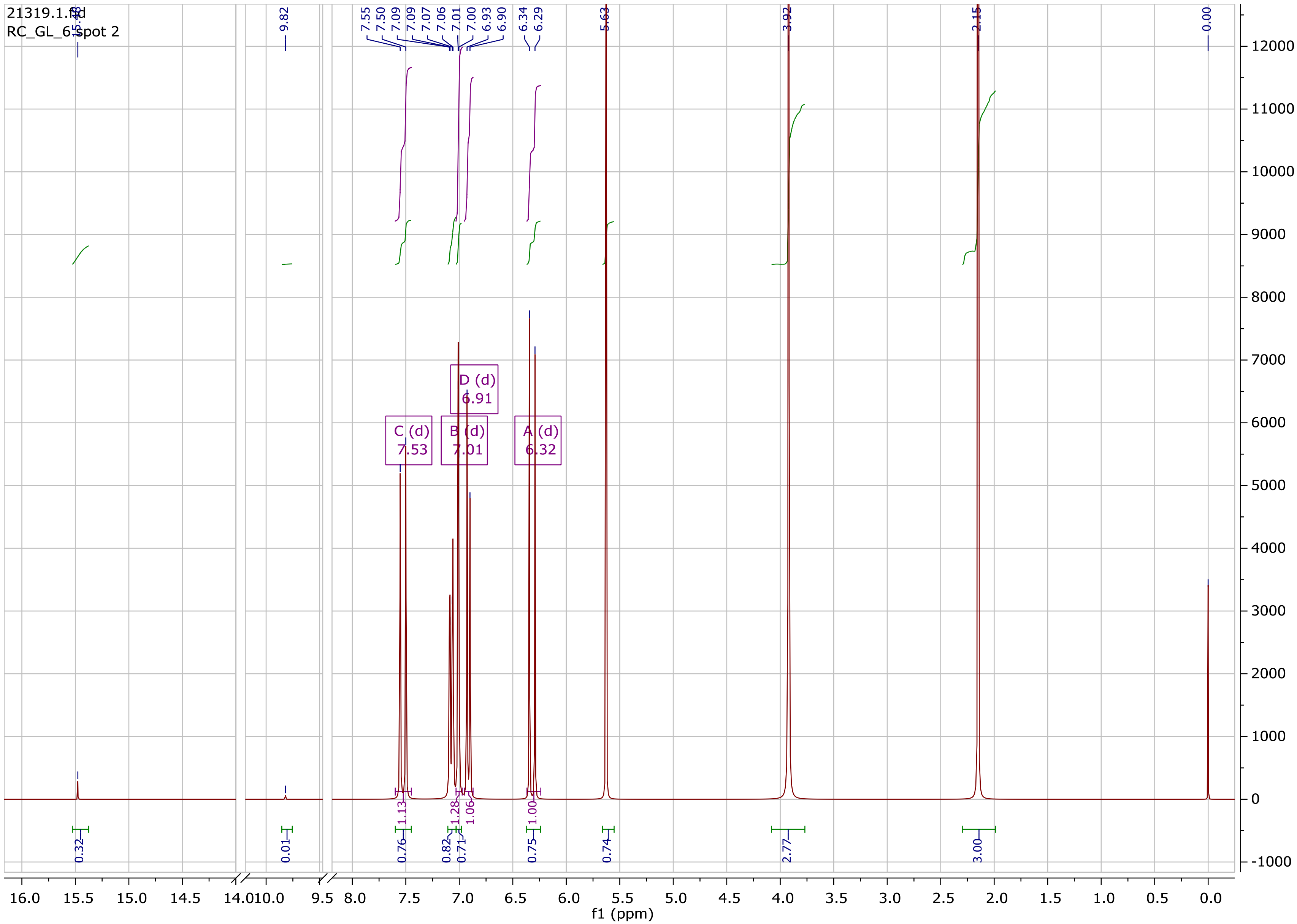

Figure 5. ^{1}H NMR spectrum of **3**.

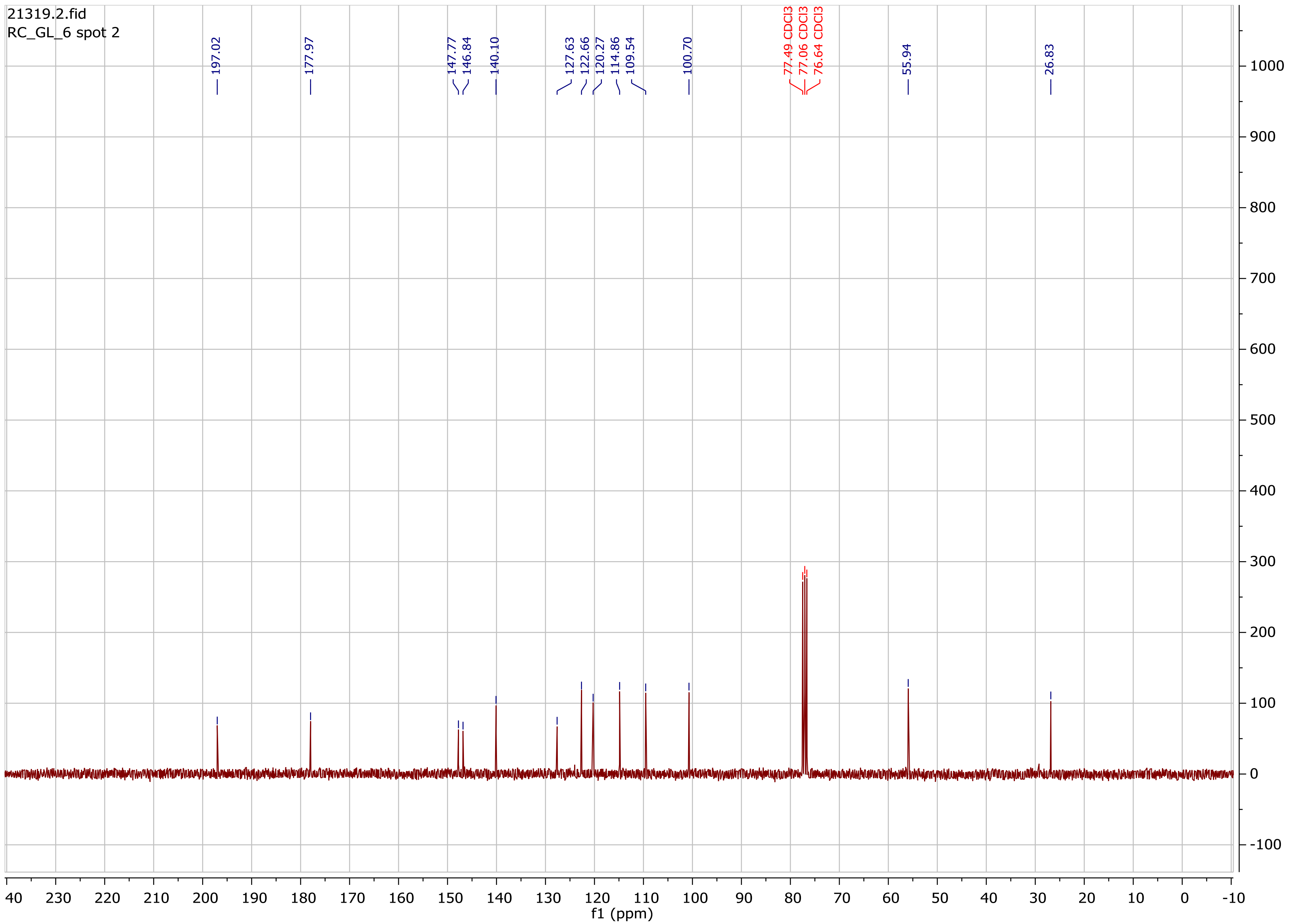

Figure 6. ^{13}C NMR spectrum of **3**.

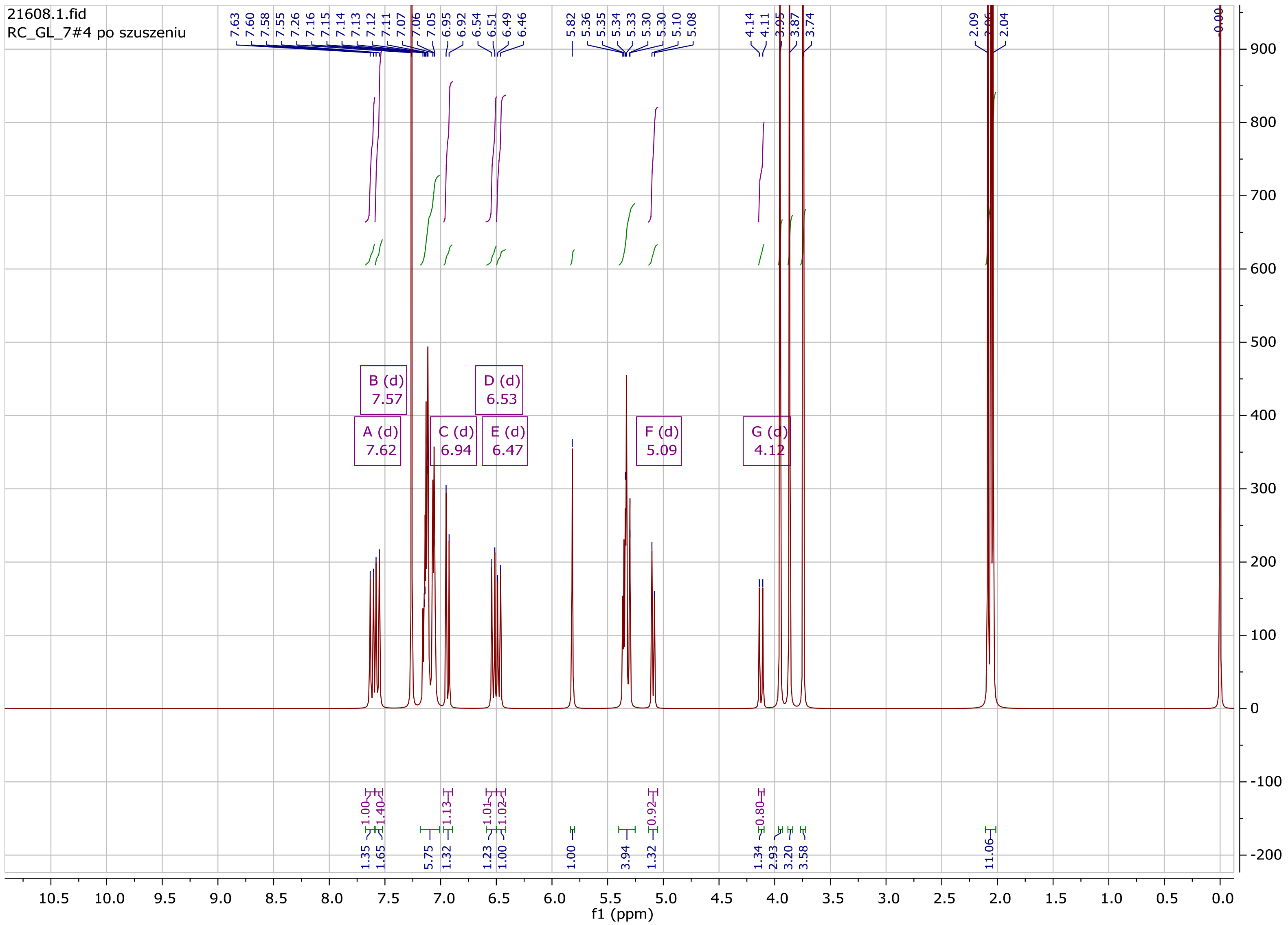

Figure 7. ^{1}H NMR spectrum of **4**.

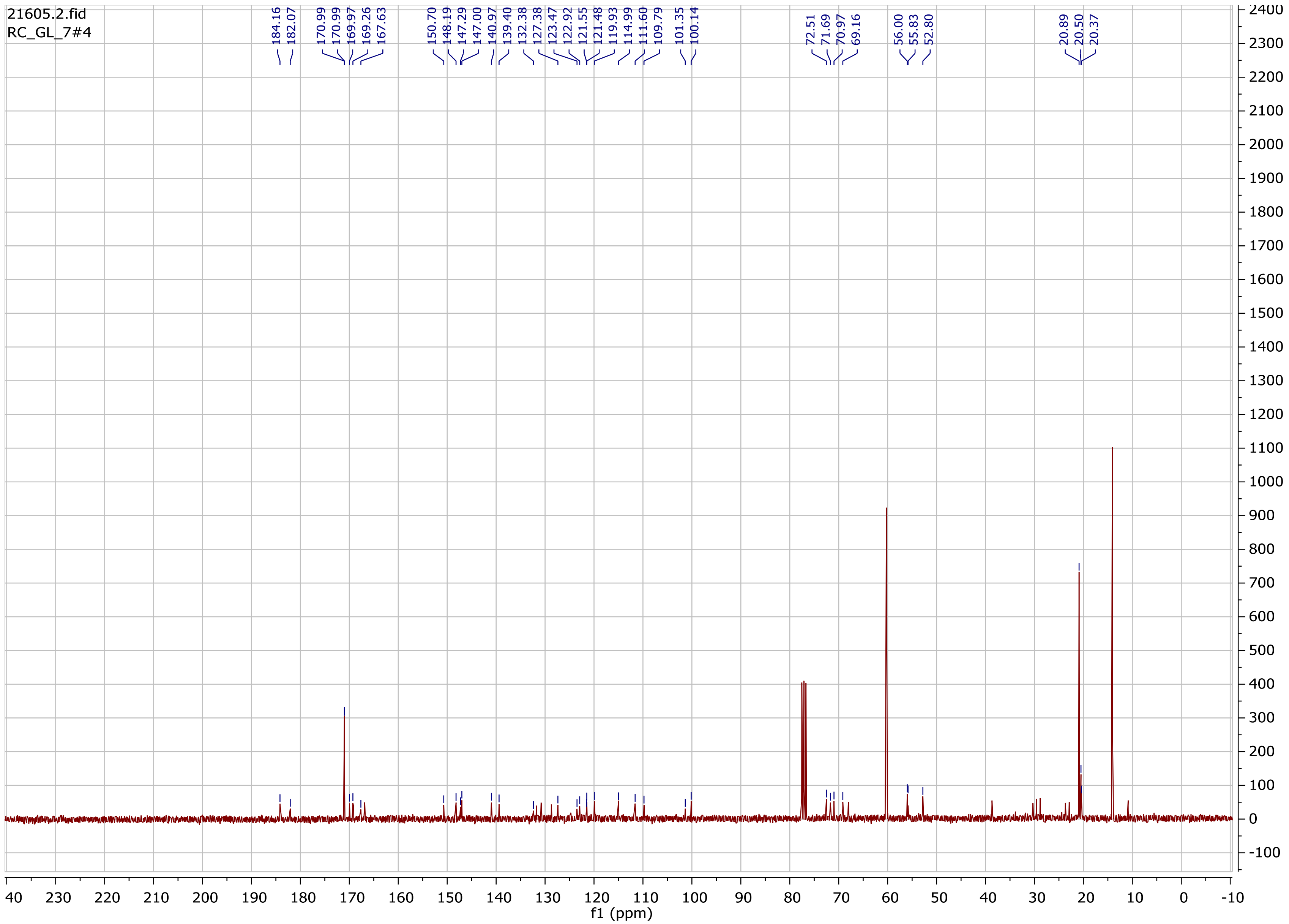

Figure 8. ^{13}C NMR spectrum of **4**.

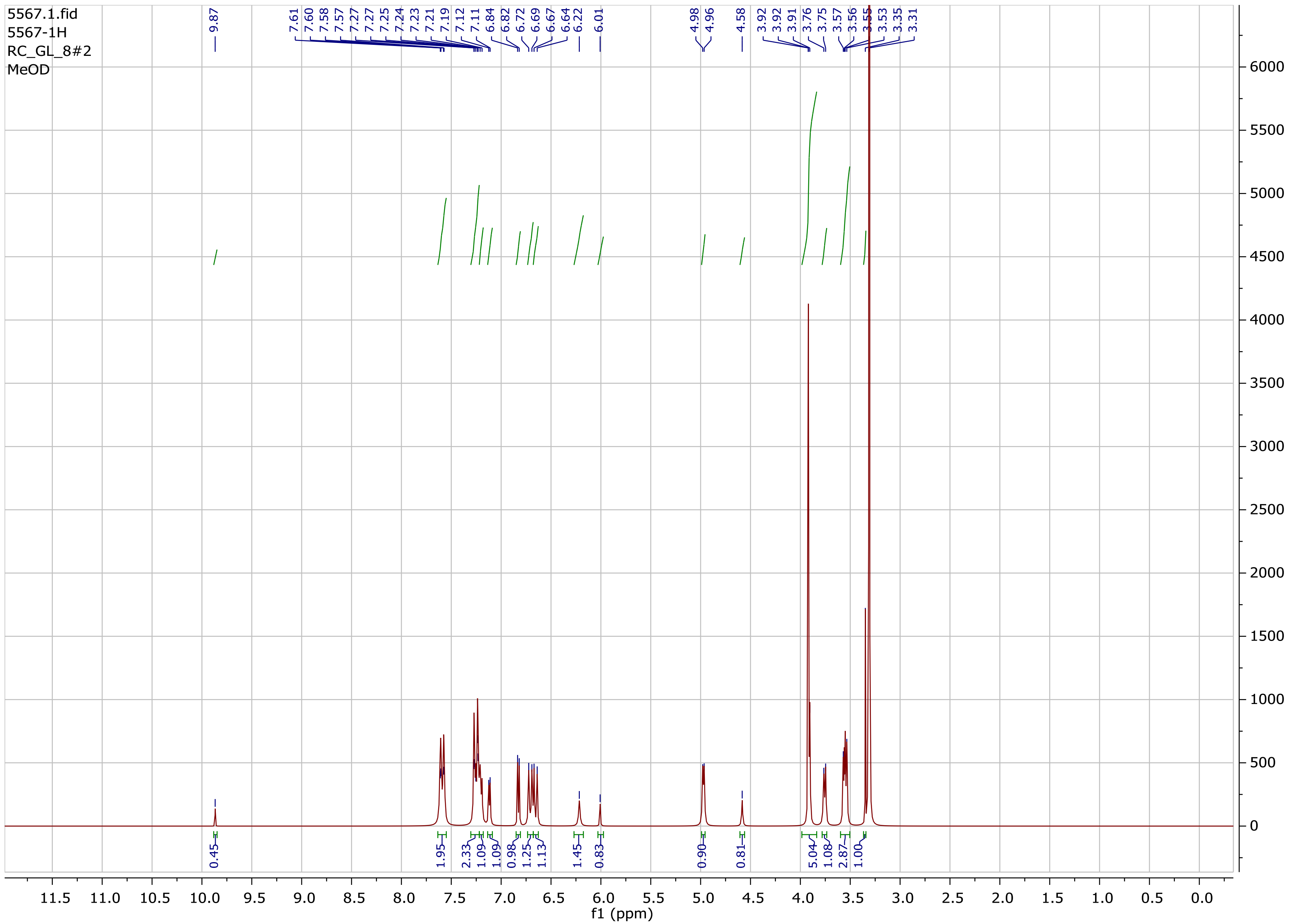

Figure 9. ^{1}H NMR spectrum of **5**.

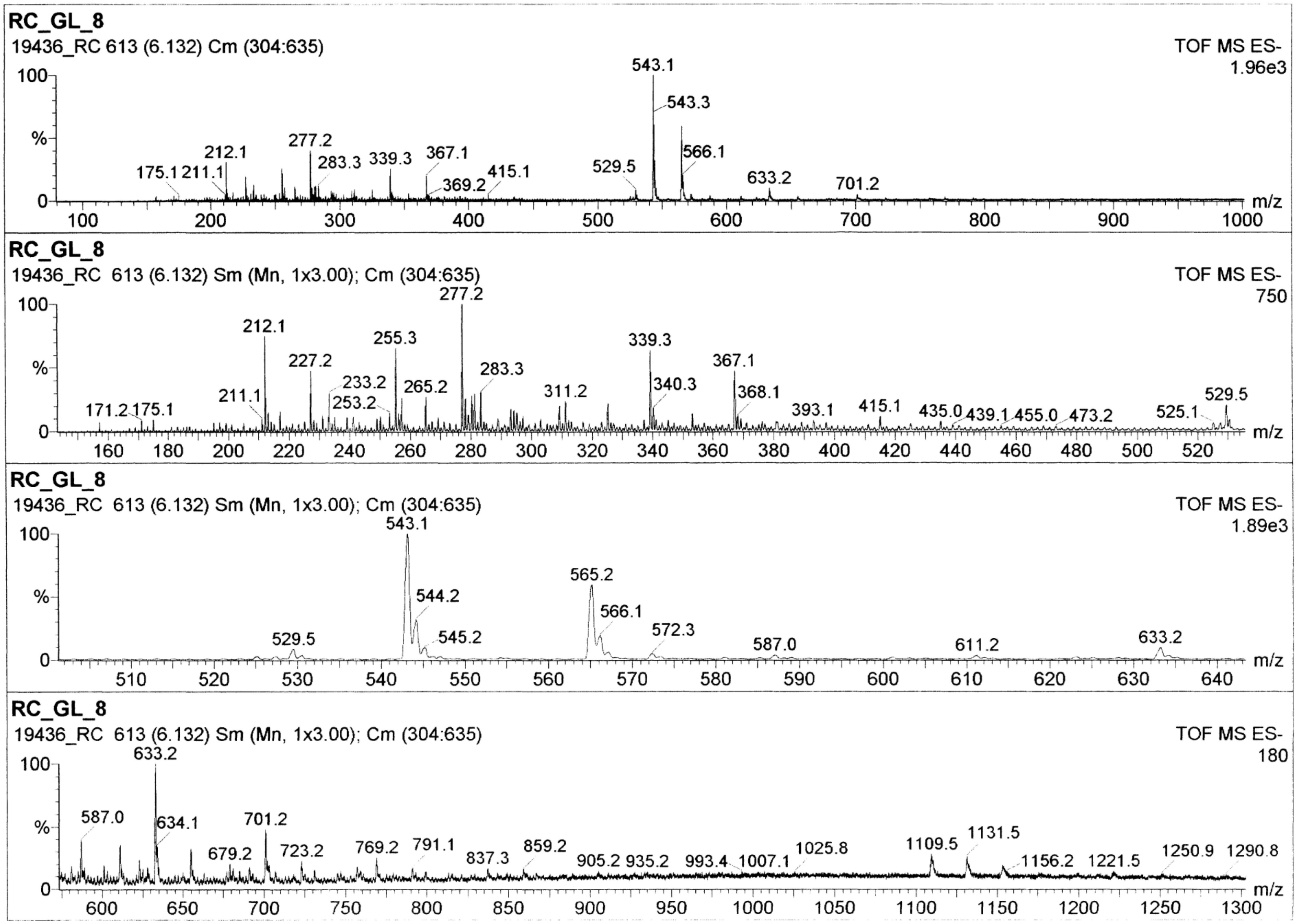

Figure 10. MS spectrum of **5**.
